# Fractional amplitude of low-frequency fluctuations associated with μ-opioid and dopamine receptor distributions in the central nervous system after high-intensity exercise bouts

**DOI:** 10.3389/fnimg.2024.1332384

**Published:** 2024-02-22

**Authors:** Henning Boecker, Marcel Daamen, Angelika Maurer, Luisa Bodensohn, Judith Werkhausen, Marvin Lohaus, Christian Manunzio, Ursula Manunzio, Alexander Radbruch, Ulrike Attenberger, Juergen Dukart, Neeraj Upadhyay

**Affiliations:** ^1^Clinical Functional Imaging Group, Department of Diagnostic and Interventional Radiology, University Hospital Bonn, Bonn, Germany; ^2^Clinical Research, German Center for Neurodegenerative Diseases (DZNE) Bonn, Bonn, Germany; ^3^Sportsmedicine, Department of Paediatric Cardiology, University Hospital Bonn, Bonn, Germany; ^4^Department of Neuroradiology, University Hospital Bonn, Bonn, Germany; ^5^Department of Diagnostic and Interventional Radiology, University Hospital Bonn, Bonn, Germany; ^6^Institute of Neuroscience and Medicine, Brain and Behaviour (INM-7), Research Centre Jülich, Jülich, Germany; ^7^Institute of Systems Neuroscience, Medical Faculty, Heinrich Heine University Düsseldorf, Dusseldorf, Germany

**Keywords:** acute exercise bout, rs-fMRI, fALFF, neurotransmitters, μ-opioidergic, dopaminergic, endocannabinoids

## Abstract

**Introduction:**

Dopaminergic, opiod and endocannabinoid neurotransmission are thought to play an important role in the neurobiology of acute exercise and, in particular, in mediating positive affective responses and reward processes. Recent evidence indicates that changes in fractional amplitude of low-frequency fluctuations (zfALFF) in resting-state functional MRI (rs-fMRI) may reflect changes in specific neurotransmitter systems as tested by means of spatial correlation analyses.

**Methods:**

Here, we investigated this relationship at different exercise intensities in twenty young healthy trained athletes performing low-intensity (LIIE), high-intensity (HIIE) interval exercises, and a control condition on three separate days. Positive And Negative Affect Schedule (PANAS) scores and rs-fMRI were acquired before and after each of the three experimental conditions. Respective zfALFF changes were analyzed using repeated measures ANOVAs. We examined the spatial correspondence of changes in zfALFF before and after training with the available neurotransmitter maps across all voxels and additionally, hypothesis-driven, for neurotransmitter maps implicated in the neurobiology of exercise (dopaminergic, opiodic and endocannabinoid) in specific brain networks associated with “reward” and “emotion.”

**Results:**

Elevated PANAS Positive Affect was observed after LIIE and HIIE but not after the control condition. HIIE compared to the control condition resulted in differential zfALFF decreases in precuneus, temporo-occipital, midcingulate and frontal regions, thalamus, and cerebellum, whereas differential zfALFF increases were identified in hypothalamus, pituitary, and periaqueductal gray. The spatial alteration patterns in zfALFF during HIIE were positively associated with dopaminergic and μ-opioidergic receptor distributions within the ‘reward' network.

**Discussion:**

These findings provide new insight into the neurobiology of exercise supporting the importance of reward-related neurotransmission at least during high-intensity physical activity.

## Highlights

- Positive mood changes, indexed as elevated PANAS Positive Affect, were identified after high- and low-intensity exercise bouts, supporting previous accounts on mood-improving effects of physical activity.
- High-intensity exercise was found to be associated with distributed changes in fractional amplitude of low-frequency fluctuations, indicating enduring neural activity changes after anaerobic exercise bouts.
- Results of spatial cross-correlations with representative PET neurotransmitter distribution maps suggest involvement of dopaminergic and opioidergic neurotransmission at least after high-intensity exercise, with weak evidence for the endocannabinoid system.
- Utilizing spatial cross-correlations of changes in fractional amplitude of low-frequency fluctuations and representative PET neurotransmitter distribution maps, despite being an indirect metric, provides an innovative methodological framework for human exercise research, as it allows for non-invasive testing of acute exercise-related changes of multiple neurotransmitter.

## Introduction

Physical activity is considered one of the most efficient mood-regulating behaviors (Thayer et al., 1994). Much work has been undertaken to unravel the underlying mechanisms of affective changes triggered by physical activity in animal models and in a growing number of human studies (Basso and Suzuki, 2017). Various studies show that moderate exercise regimens induce acute improvements of positive affect, especially immediately after exercise (Reed and Ones, 2006). Moreover, there is some evidence that acute exercise can exert motivational effects (Bothe et al., 2013), including phenomena of transient appetite suppression or altered reactivity to food cues (Dorling et al., 2018; Dera et al., 2023; Thackray et al., 2023).

Meanwhile, the underlying changes in regional brain activity remain understudied. Considering cerebral blood flow (CBF) as a frequently-used proxy for brain activity, human evidence mainly comes from transcranial doppler sonography or near infrared spectroscopy studies. They suggest transient increases in perfusion which may plateau or return to baseline, depending on duration and intensity of exercise bouts, among other factors (Mulser and Moreau, 2023). But these methods provide no (or limited) information about region-specific effects, and there are theoretical accounts which assume that resource limitations may also necessitate temporary downregulation of brain metabolism in certain brain regions (e.g., the reticular-activating hypofrontality model: Dietrich and Audiffren, 2011). Indeed, preliminary evidence from magnetic resonance imaging (MRI) studies using arterial spin labeling (ASL) indicates post-exercise CBF increases in young adults for the hippocampus (Steventon et al., 2020) or posterior insula, but also concomitant CBF decreases in the medial orbitofrontal cortex and dorsal striatum (Thackray et al., 2023). Indirect evidence for region-specific activity changes also comes from resting-state functional MRI (rs-fMRI) studies. Using blood oxygenation-dependent (BOLD) fMRI, most studies examined post-exercise changes in functional connectivity (FC), i.e., the spatiotemporal coherence of spontaneous brain activity fluctuations between different brain areas which are interpreted to reflect integrated functional brain networks (Won et al., 2021): This includes exercise-related increases of FC in sensorimotor networks (Rajab et al., 2014), or affect-reward, hippocampal, cingulo-opercular, and executive control networks (Steventon et al., 2017), but also contrary findings of FC reductions (Alfini et al., 2020). Exercise intensity may also play a moderating role (Won et al., 2021), as suggested by studies observing differential pattern of FC increases and decreases in specific rs-fMRI networks after low- or high-intensity exercise bouts (Schmitt et al., 2019, 2020). Still, rs-fMRI can provide additional brain activity-related markers, including the amplitude of low-frequency fluctuations (ALFF). It represents the voxel-level magnitude of regional BOLD fluctuations in the low-frequency range (0.01–0.08 Hz) which is assumed to be proportional to neural activity (Zang et al., 2007). Fractional ALFF (fALFF) is a normalized index of ALFF which is considered to be less sensitive to physiological noise (Zou et al., 2008). Previous data showed a close association with underlying metabolic activity (Aiello et al., 2015), making it a possible proxy for changes in regional brain activity. While one recent study (Zhang et al., 2022) examined the cross-sectional relationship between fALFF measures and cardiorespiratory fitness measures in trained athletes, studies investigating brain activity responses to acute exercise by means of fALFF are lacking.

Neurotransmitters and neuromodulators are thought to play a crucial role in mediating the mood-regulating effects of exercise, and changes in affective homeostasis, anxiety, and depression have been commonly associated with monoamine (Guszkowska, 2004), endorphin (Boecker et al., 2008), and endocannabinoid (Matei et al., 2023) neurotransmission. In addition, the motivation to exercise and to overcome physical boundaries and pain, as is particularly the case for strenuous high-intensity and/or long-duration exercise regimens, has been particularly linked to the mesolimbic dopamine reward circuit (Knab and Lightfoot, 2010; Lewis et al., 2021). As yet, most of the current knowledge on exercise-induced central neurotransmission has accumulated in animal studies (Basso and Suzuki, 2017) using either *ex vivo* brain tissue assays with high-performance liquid chromatography/mass spectrometry (Hattori et al., 1994; Gamelin et al., 2016), Western blot and immunofluorescence (Galdino et al., 2014), and autoradiography (Robison et al., 2018); or *in vivo* sampling with microdialysis (Hattori et al., 1994; Meeusen and DeMeirleir, 1995; Meeusen et al., 2001). In humans, while there is some evidence for peripheral increases of transmitters (or their metabolites) during and after exercise (Goldfarb and Jamurtas, 1997; Marques et al., 2021; Desai et al., 2022), their correlation with central neurotransmission changes is unclear, and only very few *in-vivo* studies used selective pharmacological manipulations to clarify the roles of dopaminergic (Watson et al., 2005; Roelands et al., 2008; Meeusen, 2010; Roelands and Meeusen, 2010; Klass et al., 2012), opioidergic (Allen et al., 1983; Crombie et al., 2018), and endocannabinoid (Crombie et al., 2018) neurotransmission, especially regarding their mediating role in exercise-induced affect modulation and reward-related behavior. Functional neuroimaging with positron emission tomography (PET) holds unique potential for localizing and quantifying exercise-induced neurotransmission *in vivo* after acute training sessions, or long-term changes in receptor distribution after repetitive training (Boecker et al., 2012; Boecker and Drzezga, 2016). PET ligand displacement studies allow *in vivo* monitoring of endogenous transmitter trafficking at the human whole-brain level after acute exercise bouts and, thereby, to identify the link between exercise-induced behavioral measures and endogenous neurotransmission (Boecker et al., 2008). Previous human PET studies reported endogenous dopaminergic (Ouchi et al., 2001; Sacheli et al., 2018; Ando et al., 2024) and opioidergic (Boecker et al., 2008; Hiura et al., 2017; Saanijoki et al., 2018a) transmitter release following exercise challenges, with [11C]Carfentanil PET data indicating that aerobic exercise modulates anticipatory reward processing via the μ-opioid receptor system (Saanijoki et al., 2018b), and 6-O-(2-[(18)F]fluoroethyl)-6-O-desmethyldiprenorphine ([18F]DPN) PET data showing correlations between running-induced opioid release in prefrontal and limbic/paralimbic brain structures and subjective euphoria ratings (Boecker et al., 2008). While exercise increases circulating endocannabinoid levels in humans in an intensity-dependent manner (Raichlen et al., 2013; Desai et al., 2022), to the best of our knowledge, no PET tracer studies on endocannabinoid neurotransmission changes due to acute or long-term exercise challenges were published so far.

It is important to point out that despite the genuine interest in PET displacement studies during exercise challenges, studies are hampered by limited availability, cost, and radiation exposure (Boecker et al., 2008, 2012; Boecker and Drzezga, 2016). The latter aspect is the main limiting factor preventing a comprehensive analysis of the effects of exercise on several transmitter systems at the same time. Indeed, within-subject designs are largely limited to one or maximally two neurotransmitter systems due to radiation exposure limitations, especially in healthy young subjects. Recent rs-fMRI studies have shown that spatial fALFF patterns are associated with the distribution of specific receptor systems targeted by respective PET and SPECT compounds (Dukart et al., 2021) and that this approach cannot only be used to make inferences about the status of various neurotransmitter systems (e.g., in neurological disorders), but also to detect acute changes in neurotransmission in relation to pharmacological challenges. In an extension of this latter rationale, the current study intended to investigate how varying intensities of acute exercise affect fALFF changes in brain regions linked to representative PET and SPECT neurotransmitter maps (Dukart et al., 2021; Hansen et al., 2022), thereby providing indirect markers for their involvement in acute exercise. Following up on previous work suggesting intensity-dependent effects on rs-fMRI measures (Schmitt et al., 2019, 2020), the current study examined fALFF map changes as means to investigate associated neurotransmitter effects in trained male athletes after performing a control, low-intensity (LIIE), and high-intensity (HIIE) interval exercise session. Complementing a traditional voxel-wise analysis to identify individual brain regions showing exercise-related differences in brain activity (as indicated by fALFF), we tested the spatial correlation between the observed changes across regions and the distribution of neurotransmitter systems to make inferences about the exercise-induced activation of the latter. We hypothesized specific involvement of dopaminergic, μ-opioidergic, and endocannabinoid neurotransmission, in particular for the high-intensity condition (Hiura et al., 2017; Saanijoki et al., 2018a), although a possible inverse U-curve relationship was suggested for the endocannabinoid system (i.e., changes only appearing after moderate, not heavy exercise challenges: Raichlen et al., 2013). Similar curvilinear associations were discussed for mood changes (Reed and Ones, 2006): Accordingly, increases in positive mood were already anticipated for the LIIE condition (see also Schmitt et al., 2019), while it remained open whether further increase (or even decrease) would be observed in the HIIE condition. Meanwhile, we also examined potential relationships with other neurotransmitter systems provided by the JuSpace toolbox (Dukart et al., 2021) in an exploratory manner. Considering previous human exercise studies which indicated exercise-induced effects in reward- (Bothe et al., 2013; Saanijoki et al., 2018b) and affect- (Boecker et al., 2008; Maurer et al., 2022) related brain networks, we also performed separate analyses focusing on those regions which overlap with meta-analytically defined “emotion” and “reward” networks.

## Methods

### General study design

**Figure 1:**
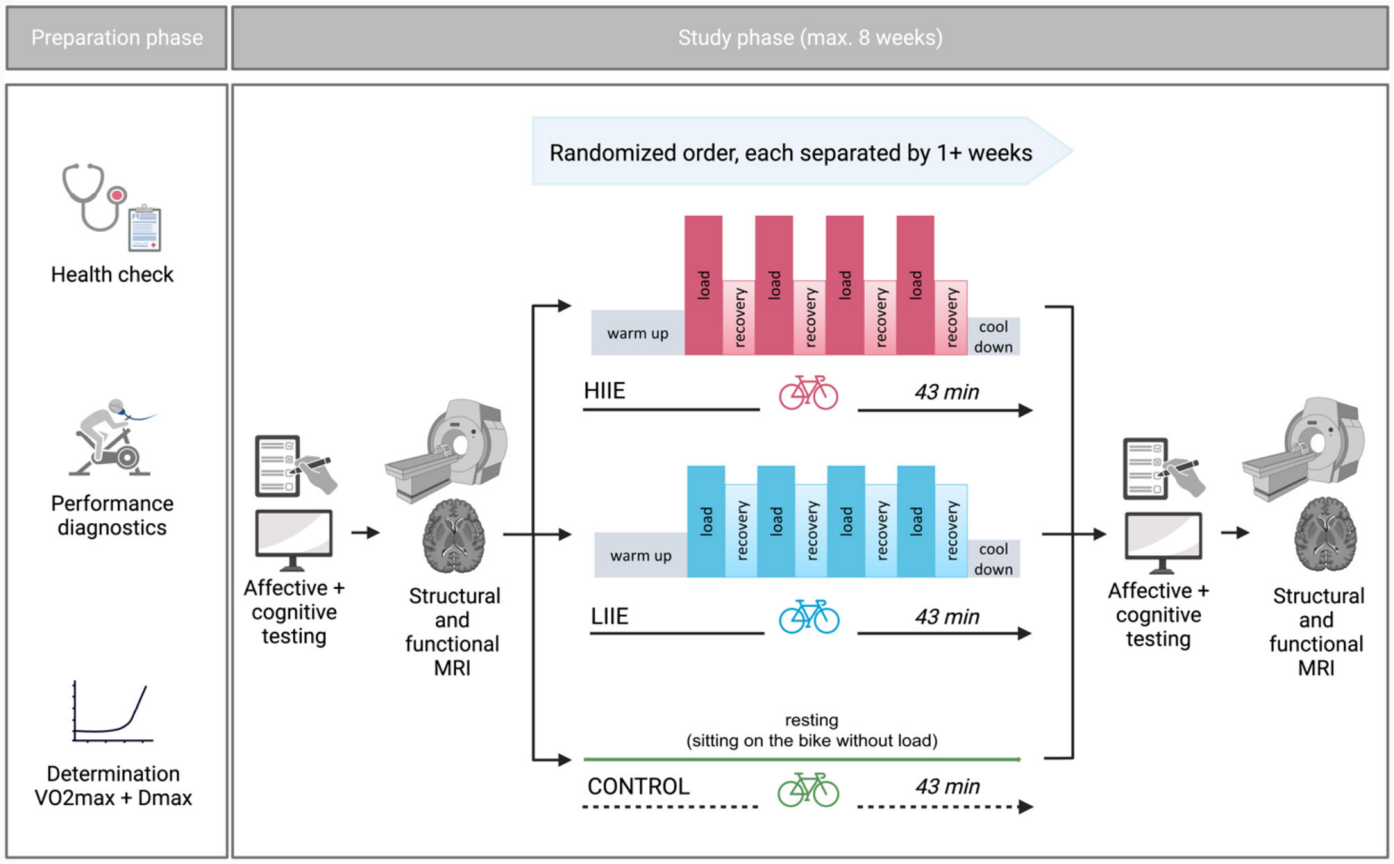
General study design (Created with Biorender.com).

This study, referred to as BEACON (“**B**icycling **E**ffects on **A**ffect and **CO**gnition in **N**euroscience”), was conceptualized as a within-subject design to investigate the acute effects of exercise bouts of differing exercise intensities on brain functional networks as well as affect and cognition (Figure 1). Some of the reported measures will also be included in other manuscripts derived from this study with different research questions.

All participants were informed about the study protocol, examinations, potential discomforts, risks and a written informed consent was obtained. Ethical approval was given by Ethics Committee at the Medical Faculty of the Rheinische Friedrich-Wilhelms-Universität Bonn (Nr. 358/19), conferring to national legislation and the Declaration of Helsinki.

Each participant completed three distinct intervention conditions—aerobic cycling (low intensity; LIIE), anaerobic cycling (high intensity; HIIE), and rest (control) condition—in a randomized order (further information below). The interventions were allowed to take place within a maximum of 8 weeks and at least 7 days apart. The night before the test, the subjects were instructed to obtain enough sleep, refrain from drinking alcohol for the preceding 24 h, and hold off on caffeine 2 h before the test. Additionally, subjects were told not to exercise 24 h beforehand. To prevent measurement differences owing to anxiety on the first exam day, subjects experienced a mock scanner session before the actual MRI. Other than that, the processes were carried out in a uniform manner on each examination day. First, subjects completed general and mood-related questionnaires as well as cognitive tests [a condensed version of the Attention Network Test (ANT) (Weaver et al., 2013) and Mnemonic Similarity Task (MST) (Stark et al., 2019) were gathered, but were not subject of the current analyses]. Then, rs-fMRI was acquired. Subsequently, one of the three interventions was performed. After the intervention, mood questionnaires, cognitive tests as well as fMRI were repeated in the same order as pre-intervention.

### Participants

Well-trained right-handed male athletes aged between 20–35 years were recruited via flier distribution at local cycling and triathlon clubs as well as the university hospital and social media. Subject enrollment was restricted to male participants in order to minimize variance due to hormonal fluctuations during the menstrual cycle. Individuals with current and/or previous severe psychiatric, neurologic or cardiovascular diseases in addition to the typical MRI-specific exclusion criteria i.e., claustrophobia, non-removable metal/implants, tattoos exceeding a critical size or other prohibiting reasons were excluded. Additionally, in order to ensure a high fitness level of participants, individuals with a relative maximum oxygen uptake (relVO_2max_) below 55 ml/min/kg were excluded (De Pauw et al., 2013).

### Experimental procedures

#### Preparation phase

##### General screening

Selection was performed prior to the study phase: It included the recording of sociodemographic characteristics, a sports medical examination and performance diagnostics. A sociodemographic questionnaire and vocabulary test were administered to obtain descriptive characteristics such as age, educational level, and estimated verbal intelligence. The International Physical Activity Questionnaire (IPAQ) (Booth, 2000) was carried out to assess the physical activities of participants in everyday life. Edinburgh-Handedness-Inventory (Oldfield, 1971) was used to assess handedness. Further, participants were screened for psychiatric symptoms using several questionnaires such as the Mini International Neuropsychiatric Interview (Sheehan et al., 1998), the Beck Depression Inventory (BDI) (Hautzinger et al., 1994), and the trait anxiety of the State-Trait-Anxiety Inventory (STAI) (Spielberger, 1983). Moreover, a substance abuse questionnaire excluded possible substance use disorders.

The medical sports examination included an anamnestic questionnaire, lung and cardiac auscultation, and a 12-lead resting ECG. Where necessary, an additional transthoracic echocardiogram was carried out.

##### Performance diagnostics

Performance diagnostics, which included a maximal incremental step test on a cycling ergometer (Cyclus2, RBM Elektronik-Automation GmbH, Leipzig, Germany), were carried out to ascertain physical fitness and, consequently, individual training intensities. The Cyclus2 made it possible to mount each participant's own bike, guaranteeing that each subject would cycle in the same unique position throughout the study. The initial workload for the incremental step test was 100 Watts (W), followed by 20 W increases every 3 min at a cadence of 80 revolutions per min (rpm) until volitional exhaustion. Power was digitally controlled with direct drive throughout the performance diagnostics and the ensuing exercise modifications. When the cadence dropped to < 65 rpm, the test was stopped.

Throughout the test, measurements of oxygen uptake (VO_2_), the metabolic respiratory quotient (RER), heart rate (HR) (Cortex meta-analyzer 3B, Leipzig, Germany, and Polar Electro Oy, Kempele, Finland), and the electrocardiogram (ECG) (Cardio 100 USB, Ergoline GmbH, Bitz, Germany) were continually recorded. A validated H10 chest strap was used to continually measure HR variability. Additionally, blood pressure was checked at each step. The final 15 seconds of each procedure were used to draw 20 μl of capillary blood from the earlobe, mix it immediately with 1 mL hemolysis solution, and analyze the sample amperometrically and enzymatically using an EBIOplus system (EKF Diagnostic Sales, Magdeburg, Germany). Within the last 15 seconds of each increment, the rating of perceived exertion (RPE) was measured using the Borg scale (6 to 20 points: 6 - no exertion at all, 20 - maximal exertion) (Borg, 1998).

Exhaustion was considered when at least two of the following criteria were met: plateauing in VO_2_, RER ≥ 1.05, high levels of blood lactate (≥8 mmol/L), a RPE of ≥18. The greatest 30-second moving average of VO_2_ divided by body mass (mL/min/kg) was used to calculate relVO_2max_. Those who had a relVO_2max_ of < 55 mL/min/kg were not included in the study.

#### Study phase

##### Intervention

Following the first MRI examination, volunteers completed the supervised intervention on a bicycle ergometer. Depending on the treatment condition, subjects performed one of the two alternative exercise intensities (low and high) or the control condition (without load) in randomized order. An adapted Latin Square was used for randomization: ABC, ACB, BCA, BAC, CBA, CAB; order of these combinations additionally randomized within blocks of 6 subjects. A 4^*^4-min load was alternated with 3 min of active recovery during the exercise interventions. Individual exercise intensities were established using the incremental step test. In the exercise interventions, subjects began with a 10-min warm-up at 1,5 Watts per kilogram of body weight (W/kg BW) followed by interval training and a final cooldown of 5 min < 1,5 W/kg BW. The intervention intensities were established as follows: (i) LIIE: 4 ^*^ 4 minutes' load at 100% first rise (W) with 3 min active recovery at 90% of first rise; (ii) HIIE: 4 ^*^ 4 min' load at 110% D_max_ (W) with 3 min active recovery at 60% of D_max_; and (iii) control: no load while sitting on the cycling ergometer for 43 min.

The modified D_max_ approach of Zwingmann et al. (2019) was used to calculate lactate thresholds. The first rise was defined as the moment at which the lactate concentration increased by >4% from the previous value. The last 30 seconds of each load and recovery period were used to measure blood lactate, HR_int_ (during the intervention), and RPE. Blood pressure was tested both before and after the treatment.

##### Physiological and psychological testing

Each study day began with the subjects filling out a special questionnaire that measured their current levels of exhaustion, discomfort, previous night's sleep quantity and quality, and recent caffeine and alcohol intake.

To account for physiological effects due to exhaustion, the corresponding MoodMeter (Kleinert, 2006; Wollseiffen et al., 2016) subscale (consisting of the items “feeble” and “drowsy” from psychological strain), was included in the statistical analyses as this may influence mood and fMRI outcomes.

The Positive And Negative Affect Schedule (PANAS) was one of the questionnaires used to gauge mood in general (Krohne et al., 1996), which served as the primary affect measure in the presented analysis. Participants had to rate their current mood state based on a total of 20 adjectives, 10 of which are assigned to the dimension of positive affect and 10 to the dimension of negative affect (scoring with 5-point Likert scale ranging from 1 “not at all” to 5 “very much”). This way, the questionnaire allowed for a separate dimensional assessment of pleasure and displeasure effects which are assumed to be partially dissociable on the brain system level (Norris et al., 2010), and may therefore show differential susceptibility to acute exercise. To examine potential anxiolytic effects of exercise more specifically, the STAI-State scale was also collected (Spielberger, 1983).

##### MRI acquisition

On each study day, participants completed two identical MR sessions. To account for potential influences of circadian rhythms on the behavioral/imaging data, measurements of each subject were always performed at the same time of the day, either from 08:00–11:00, 11:00–14:00, or 14:00–17:00. The respective MRI scans (in total six scans) were acquired on a Philips Ingenia Elition 3.0T scanner with a 32-channel head coil at the Parent-Child Center of the University Hospital Bonn.

A regular session consisted of a T1-weighted (T1w), a fieldmap and a rs-fMRI sequence. For rs-fMRI, the light in the examination room was switched off and participants were instructed to close their eyes, not to think of anything in particular, and to stay awake during the whole scan. An echo-planar imaging (EPI) protocol with blood-oxygen-level-dependent (BOLD) contrast and 3D acquisition was performed with the following specifications: TR = 1,020 ms, TE = 30 ms, acquired voxel size = 2.5 × 2.5 × 2.5 mm, reconstructed voxel size = 2.17 × 2.17 × 2.5 mm, FoV = 208 × 208 mm, flip angle = 52°, SENSE: 2, MB factor: 3, EPI factor: 41, matrix: 84 × 82, slices: 51, scan order: FH (ascending). Over a total duration of 10:04 min, 585 dynamic scans were acquired. The anatomical T1w sequences were acquired with the following specifications: TR = 10 ms, TE = 4.7 ms, acquired voxel size = 0.7 × 0.7 × 0.7 mm, reconstructed voxel size: 0.49 × 0.49 × 0.57 mm, FoV = 250 × 250 mm, flip angle = 8°. The total duration of this sequence was 6:19 min. Field mapping was performed with the following parameters: TR = 650 ms, TE = 7 ms, voxel size: 3.75 × 3.75 × 4 mm, flip angle 80°.

During all fMRI scans, HR was continuously recorded (HR_rest_) by a pulsoxymetry sensor on the index finger.

### Physiological and psychological data analysis

The analysis of the behavioral and the physiological data was performed using IBM SPSS (Statistics Version 27.0. IBM Corp. Armonk, NY).

#### Exercise intervention

To evaluate whether exercise intensity between the low and high condition differed significantly, paired *t*-tests with Bonferroni correction were performed for the variables HR_int_, lactate concentration and RPE. Significance was considered at *p* < 0.05 and the effect size is reported as Cohen's d.

#### HR_**rest**_ and exhaustion

Values above or below the mean ± 2.5 standard deviations (SD) were removed from the data set as outliers for HR_rest_. Statistical analysis of the outlier-corrected means of HR_rest_ and raw exhaustion scores was performed using a 2 (timepoint: pre/post) × 3 (condition: control, low, high) repeated measures ANOVA, separately. *Post-hoc* paired *t-*tests with Bonferroni correction were also performed. Significance was considered at *p* < 0.008 (0.05/6), and the effect size was reported as partial eta square and Cohen's d.

#### Mood questionnaires

A repeated measures 2 (timepoint: pre-intervention/post-intervention) × 3 (condition: control/low/high) ANOVA was performed to assess the changes in PANAS and STAI-State according to the conditions. *Post-hoc* paired *t*-tests with Bonferroni correction were also performed. Significance was considered at *p* < 0.008 (0.05/6), and the effect size was reported as partial eta square and Cohen's d.

### MRI data analysis

#### Quality control and image preprocessing

Resting-state fMRI data underwent a well-recognized MRIQC (Esteban et al., 2017) pipeline for visual quality check regarding acquisition artifacts. No datasets with substantial quality issues were identified.

Resting-state fMRI data were pre-processed using the fmriprep pipeline (https://fmriprep.org/en/stable/) (Esteban et al., 2019). Preprocessing started with skull stripping the fMRI data using a custom fmriprep methodology. Furthermore, fieldmaps were used to correct for susceptibility distortions by applying the FSL 6.0 (Jenkinson et al., 2012) fugue (https://fsl.fmrib.ox.ac.uk/fsl/fslwiki/FUGUE) and SDCflows tools (Wang et al., 2017). The estimated susceptibility distortion was used to create a corrected BOLD EPI reference in order to accurately register the functional data to the anatomical reference. The BOLD EPI reference was registered to the T1-weighted reference using a boundary-based registration approach. Before spatiotemporal filtering, head motion parameters with respect to BOLD EPI reference were estimated using mcflirt (Jenkinson et al., 2002). A single composite transform was applied to the BOLD time-series to correct for head motion and susceptibility distortions, and resampled to their original native space. Thereafter, BOLD time series were normalized to standard MNI152NLin2009cAsym space (Fonov et al., 2011). A detailed description of the fmriprep pipeline can be found in the Supplementary material [“Detailed Description of the fMRIPrep Pipeline (Boilerplate)”].

After preprocessing, data quality was further determined on the basis of the fmriprep DVARS and framewise displacement (FD) metrices. Both DVARS and FD reflect the rate of change of the BOLD signal across the whole brain at each frame of data and the head movement of the specific frames, respectively. In the case of more than 60% of the 585 volumes with FD larger than 0.2 mm, subjects were removed from the final analysis (Parkes et al., 2018).

Then, output functional maps were used to calculate fALFF for all six sessions (pre- and post-intervention in each condition). The 3dRSFC function in AFNI (https://afni.nimh.nih.gov/pub/dist/doc/program_help/3dRSFC.html) was applied, including quadratic detrending, band-pass filtering (0.01–0.08 Hz), 4 mm smoothing and regressing out white matter and cerebrospinal fluid signal as well as the 24 motion parameter time courses in a single step (Taylor and Saad, 2013). Normalized fALFF (zfALFF) maps were created by dividing the map through its mean.

#### Voxel-wise analyses of zfALFF maps

In a basic analysis, we compared the zfALFF maps from the pre- and post sessions of the three conditions using a voxel-wise approach, performing a 2 (timepoint: pre/post) × 3 (condition: control, low, high) repeated measures analysis of variance (ANOVA) for whole brain in SPM12 (https://www.fil.ion.ucl.ac.uk/spm/software/spm12/).

To exclude a direct effect of HR_Rest_ and Exhaustion on the zfALFF, regression analyses were performed with the zfALFF maps within each condition (HIIE, LIIE, and control) for the post-pre changes. Masks were created by saving significant clusters found at a lenient threshold of *p* < 0.01 (uncorrected). These masks from each condition were merged to one mask indicating brain regions influences by HR_Rest_ and Exhaustion. Later, the merged mask was used for exclusive masking in the repeated measure ANOVA for zfALFF.

We applied a voxel-wise cluster-defining threshold of *p* < 0.001 (uncorrected) and reported significant clusters at *p* < 0.05 after implementing a cluster-wise extent threshold (≥k) for different contrasts using the family-wise error (FWE) method for multiple comparisons correction.

#### Associations between fALFF changes and PANAS

To examine possible associations between the observed intervention-related fALFF changes and corresponding affective changes, we performed follow-up regression analyses within those brain areas which showed significant fALFF effects in the ANOVA analyses, using the questionnaire pre-to-post difference scores as the independent variable, and the corresponding pre-to-post differences of fALFF scores as the dependent variable.

#### Spatial correlations of fALFF changes with neurotransmitter maps

The JuSpace toolbox (https://github.com/juryxy/JuSpace; version 1.4) was used to compute spatial cross-correlations between the zfALFF changes per exercise condition (within-subject pairwise zfALFF differences between the pre- and post-intervention fMRI scans; computing option 6 of the toolbox) and neurotransmitter maps.

First, we tested for spatial correlations of the zfALFF changes induced by either of the three conditions (LIIE, HIIE, or control) with different neurotransmitter systems using a whole brain approach, using 14 neurotransmitter maps included in the JuSpace toolbox. In case of multiple maps per neurotransmitter system, the ones with highest reliability based on sample size and/or signal-to-noise ratio were chosen. We included the following neurotransmitter maps: dopamine synthesis, storage and transport [Fluorodopa (García-Gómez et al., 2018); DAT (Dukart et al., 2018)] and receptors [D1 (Kaller et al., 2017); D2 (Sandiego et al., 2015; Hansen et al., 2022)], GABA (gamma-aminobutyric acid) receptor (Dukart et al., 2018), endocannabinoid receptor (CB1) (Laurikainen et al., 2019; Hansen et al., 2022), μ-opioidergic receptor (MOR) (Kantonen et al., 2020; Hansen et al., 2022), serotonin receptors [5-hydroxytryptamine (5-HT1a, 5-HT1b, and 5-HT2a)] and transporter (SERT) (Savli et al., 2012), noradrenaline transporter (NET) (Hesse et al., 2017), vesicular acetyl choline transporter (VAChT) (Aghourian et al., 2017; Hansen et al., 2022), and the metabotropic glutamate receptor type 5 (mGluR_5_) (Smart et al., 2019; Hansen et al., 2022).

Second, we tested for spatial correlations of the zfALFF changes in those regions of the neurotransmitter maps which overlapped with *a priori* defined “reward” and “emotion” networks. For this hypothesis-driven approach, we focused on three pre-informed neurotransmitter systems (i.e., D2, Mu and CB1) already known to be involved in affect and reward modulation in general (Schott et al., 2008; Mechoulam and Parker, 2013; Kantonen et al., 2021), or in the exercise context more specifically (Boecker et al., 2008; Saanijoki et al., 2018b; Forteza et al., 2021; Gorrell et al., 2022; Kantonen et al., 2022). For these analyses, spatial correlations were restricted to brain regions within the neurotransmitter-specific maps which are also linked with emotion and reward processing, respectively, based on independent neuroscientific evidence: A “reward network” mask was created using a term-based meta-analysis of functional neuroimaging studies implemented in Neurosynth (https://neurosynth.org) including the ventral striatum, thalamus, cingulate, orbitofrontal cortex. An “emotion network” mask was applied in an identical manner as in our previous study (Schmitt et al., 2020) and included anterior/middle/posterior cingulate cortex, inferior/medial/middle/superior orbitofrontal cortex, dorsolateral prefrontal cortex, hypothalamus, insula, amygdala, nucleus accumbens, and pallidum. Both masks are displayed in the Supplementary Figure S1.

Spearman correlation coefficients were calculated between the single subject pairwise zfALFF difference maps and the respective neurotransmitter maps, after parcellation according to the Neuromorphometric atlas (Landman and Warfield, 2012). Exact permutation-based *p*-values (with 10.000 permutations) were computed for all comparisons to test for significance of the mean correlation coefficients observed across participants from the null distribution. Finally, we reported the spatial cross-correlations at *p* < 0.05, false discovery rate (FDR) corrected for multiple comparisons.

#### fALFF-neurotransmitters correlations with mood questionnaires

Finally, we used Spearman correlations to assess the relationships between the outputs of JuSpace (significant zfALFF-neurotransmitters correlation, Fisher's Z-transformed) and the PANAS and the STAI-State, respectively. Significant results were reported at *p* < 0.05 after correcting for multiple comparisons using Bonferroni correction.

## Results

### Demographic measures

Among twenty-nine recruited participants only twenty (age: 27.3 ± 3.55 years) were included in the present analyses. Reasons for exclusion were: injury during private exercise or illness (*N* = 4), failure to reach the specified VO_2max_ limit of 55 mL/min/kg (*N* = 3), and motion artifact in MRI (*N* = 2). An overview of demographic and physiological characteristics of the sample is provided in Table 1 and in other manuscripts from this work. According to the IPAQ, *N* = 19 subjects could be categorized as highly active and *N* = 1 as moderately active.

**Table 1:** Demographic and physiological characteristics.

**Measures**	**Values (Mean ±SD)**
Age (years)	27.3 ± 3.6
Weight (kg)	76.3 ± 6.5
Height (cm)	181.6 ± 6.3
BMI (kg/m^2^)	23.1 ± 1.1
VO_2_max (mL/min/kg)	58.5 ± 3.5
Education	18.6 ± 2.1
Estimated IQ	108.1 ± 5.8
BDI	1.6 ± 1.5
STAI-Trait	28.7 ± 3.5
EHI	76.9 ± 22.4

BMI, body mass index; IQ, intelligent quotient; BDI, Beck Depression Inventory; STAI, State-Trait Anxiety Inventory; EHI, Edinburgh Handedness Inventory, Lateralization Quotient.

### Physiological data

Results of the HR_int_ and HR_rest_ are also reported in other manuscripts from this study (in preparation/parallel submission) with different research questions. Since the intervention is a key component of the study, overlapping reporting is inevitable.

#### Exercise intervention

All included participants were able to train within the desired power output in the LIIE and the HIIE interventions. Statistical analysis revealed significant differences between the two exercise conditions. Values and statistics of HR_int_ (Supplementary Table S1), lactate concentration (Supplementary Table S2), RPE (Supplementary Table S3), and the actual power values during the intervention (Supplementary Table S4) are summarized in the Supplementary material.

#### Heart rate during fMRI (HR_**rest**_)

The results of the repeated measures ANOVA following Greenhouse-Geisser method for sphericity correction showed a significant main effect of condition [F_(2, 32.36)_ = 6.80, *p* = 0.005, η^2^ = 0.26], a main effect of time [F_(1, 19)_ = 7.02, *p* = 0.016, η^2^ = 0.27], and a significant time × condition interaction [F_(2, 26.06)_ = 18.01, *p* < 0.001, η^2^ = 0.49] for the variable HR_rest_.

*Post-hoc* tests showed significantly increased HR_rest_ during the scan from pre to post HIIE [t_(19)_ = 4.71, *p* < 0.001, d = 1. 054; high pre: 52.03 ± 6.64 bpm; high post: 58.99 ± 6.95 bpm] and a significantly decreased HR_rest_ from pre to post of the control condition [t_(19)_ = −5.38, *p* < 0.001, d = −1.20; control pre: 52.49 ± 8.37 bpm; control post: 48.73 ± 9.01 bpm]. No significant HR_rest_ changes were detected from pre to post LIIE [t_(19)_ = 1.65, *p* = 0.116, d = −0.37; low pre: 52.44 ± 7.71 bpm; low post: 54.76 ± 9.49 bpm]. Comparing the conditions with each other, significant results were found comparing the HIIE vs. the control condition [“high post minus pre” vs. “control post minus pre”; t_(19)_ = 6.85, *p* < 0.001, d = 1.531; delta high: 6.96 ± 6.60 bpm; delta control: −3.75 ± 3.12 bpm] and comparing the LIIE vs. the control condition (“low post minus pre” vs. “control post minus pre”; t_(19)_ = 4.50, *p* < 0.001, d = 1.01; delta low: 2.32 ± 6.30 bpm; delta control: −3.75 ± 3.12 bpm). Results were not significant comparing the HIIE vs. LIIE condition [“high post minus pre” vs. “low post minus pre”; t_(19)_ = 2.01, *p* = 0.059, d = 0.45; delta high: 6.96 ± 6.60 bpm; delta low: 2.32 ± 6.30 bpm].

#### Exhaustion

Repeated measures ANOVA showed a significant main effect of time [F_(1, 19)_ = 4.61, *p* = 0.045, η^2^ = 0.19], and main effect of condition [F_(2, 38)_ = 3.42, *p* = 0.043, η^2^ = 0.153], but no significant time × condition interaction [F_(2, 38)_ = 1.05, *p* = 0.361, η^2^ = 0.052] for the variable exhaustion. *Post-hoc* analysis revealed no significant effects after Bonferroni correction: HIIE condition [t_(19)_ = 2.13, *p* = 0.046, d = 0.48; high pre: 1.65 ± 1.33; high post: 1.07 ± 1.02]; LIIE condition [t_(19)_ = 1.453, *p* = 0.163, d = 0.32; low pre: 1.02 ± 1.21; low post: 0.62 ± 0.76]; control condition [t_(19)_ = 0.322, *p* = 0.751, d = 0.072; control pre: 1.27 ± 1.22; control post: 1.20 ± 1.13].

### Behavioral data

#### PANAS

**Figure 2:**
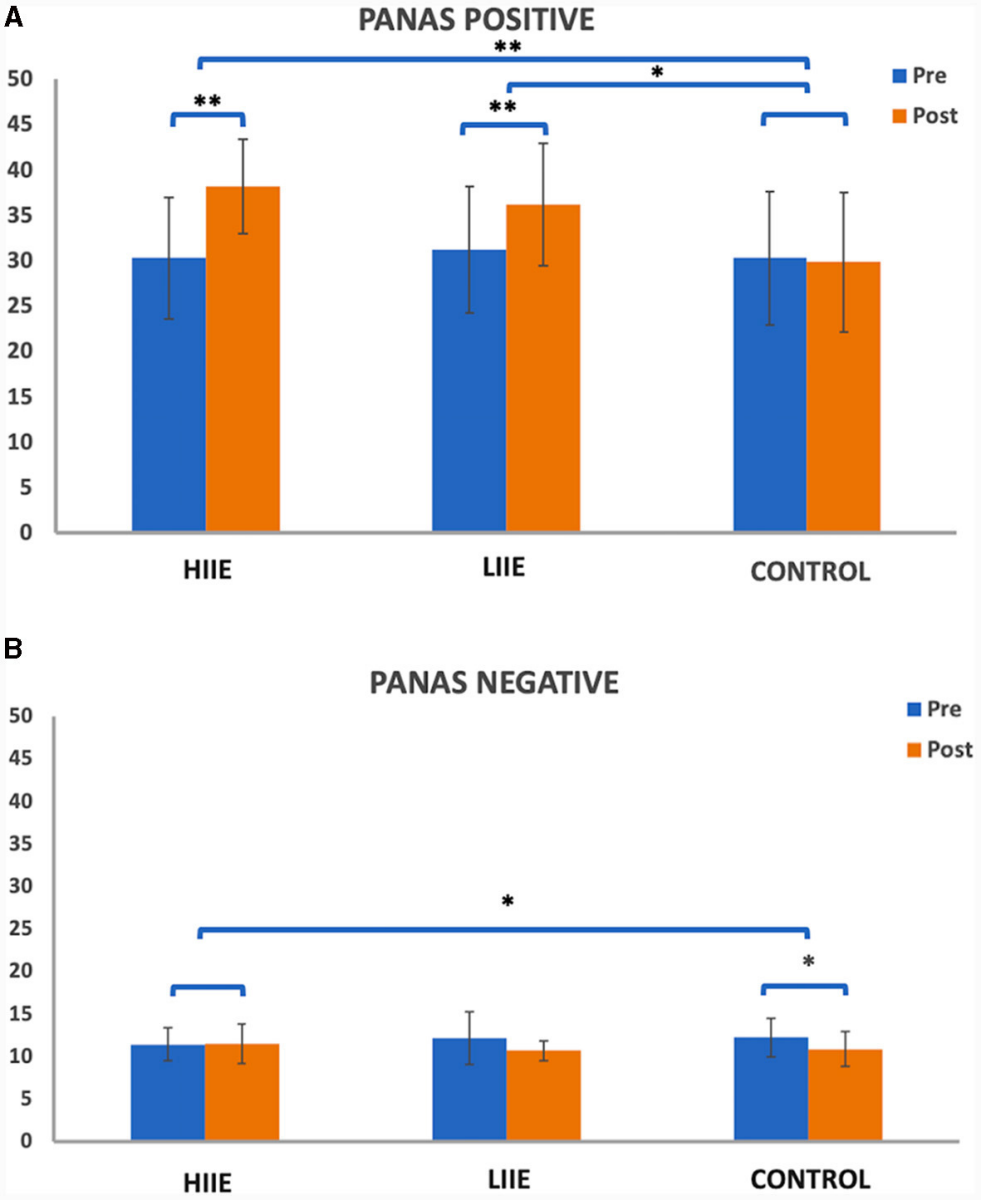
Representation of the PANAS **(A)** positive and **(B)** negative scales pre- and post conditions. Whiskers represent the standard deviation. *p < 0.008 (Bonferroni-corrected); **p < 0.001.

A 2 × 3 repeated measures ANOVA for PANAS Positive Affect found significant main effects of condition [F_(2, 38)_ = 17.29, *p* < 0.001, η^2^ = 0.48] and time [F_(1, 19)_ = 28.17, *p* < 0.001, η^2^ = 0.60], as well as a time × condition interaction [F_(2, 38)_ = 17.08, *p* < 0.001, η^2^ = 0.47]. *Post hoc* comparisons showed significant increases after HIIE [t_(19)_ = 6.42, *p* < 0.001, Cohen's d = 1.43; high pre: 30.30 ± 6.67; high post: 38.20 ± 5.21] and after LIIE [t_(19)_ = 5.67, *p* < 0.001, Cohen's d = 1.27; low pre: 31.25 ± 6.97; low post: 36.25 ± 6.76]. No significant differences were observed from pre to post control conditions. Comparing the conditions with each other, significant results were found when testing HIIE vs. the control condition [“high post minus pre” vs. “control post minus pre”; t_(19)_ = 7.12, *p* < 0.001, Cohen's d = 1.59; delta high: 7.90 ± 5.50; delta control: −0.45 ± 5.73] and testing LIIE vs. the control condition [“low post minus pre” vs. “control post minus pre”; t_(19)_ = 3.38, *p* = 0.003, Cohen's d = 0.756; delta low: 5.0 ± 3.95; delta control: −0.45 ± 5.73]. Testing HIIE vs. the LIIE condition showed no significant differences [“high post minus pre” vs. “low post minus pre”; t_(19)_ = 1.89, *p* = 0.073, Cohen's d = 0.42; delta high: 7.90 ± 5.50; delta low: 5.0 ± 3.95] (see Figure 2A).

On the other hand, the 2 × 3 repeated measures ANOVA for the PANAS Negative Affect scale showed a significant main effect of time [F_(1, 19)_ = 9.68, *p* = 0.006, η^2^ = 0.34] and a significant time x condition interaction [F_(2, 38)_ = 5.11, *p* = 0.011, η^2^ = 0.21] but no significant main effect of condition [F_(2, 38)_ = 0.099, *p* = 0.906, η^2^ < 0.01]. *Post-hoc* tests showed significantly decreased PANAS Negative from pre to post control condition [t_(19)_ = −3.94, *p* = 0.001, d = −0.882; control pre: 12.20 ± 2.62; control post: 10.85 ± 2.03]. No significant differences were observed from pre to post of the LIIE condition [t_(19)_ = −2.43, *p* = 0.025, d = −0.544; low pre: 12.10 ± 3.09; low post: 10.65 ± 1.14] or from pre to post HIIE [t_(19)_ = −0.37, *p* = 0.716, d = −0.083; high pre: 11.40 ± 1.93; high post: 11.50 ± 2.33]. Comparing the conditions with each other, significant results were found only comparing the high-intensity vs. the control condition [“high post minus pre” vs. “control post minus pre”; t_(19)_ = 3.45, *p* = 0.003, d = 0.7721; delta high: 0.10 ± 1.21; delta control: −1.35 ± 1.531]. No significant changes were detected when testing the LIIE vs. the control condition [“low post minus pre” vs. “control post minus pre”; t_(19)_ = 0.177, *p* = 0.862, Cohen's d = 0.039; delta low: −1.45 ± 2.67; delta control: −1.35 ± 1.531] or the HIIE vs. the LIIE [“high post minus pre” vs. “low post minus pre”; t_(19)_ = 2.49, *p* = 0.022, Cohen's d = 0.56; delta high: 0.10± 1.21; delta low: −1.45 ± 2.67] (see Figure 2B).

#### STAI-state

A 2 × 3 repeated measures ANOVA for the STAI-State found no significant main effect of condition [F_(2, 38)_ = 0.055, *p* = 0.947, η^2^ < 0.01] and time [F_(1, 19)_ = 3.99, *p* < 0.060, η^2^ = 0.17], as well as no time × condition interaction [F_(2, 38)_ = 1.67, *p* = 0.20, η^2^ = 0.008].

### MR data

#### Voxelwise changes in zfALFF

**Figure 3:**
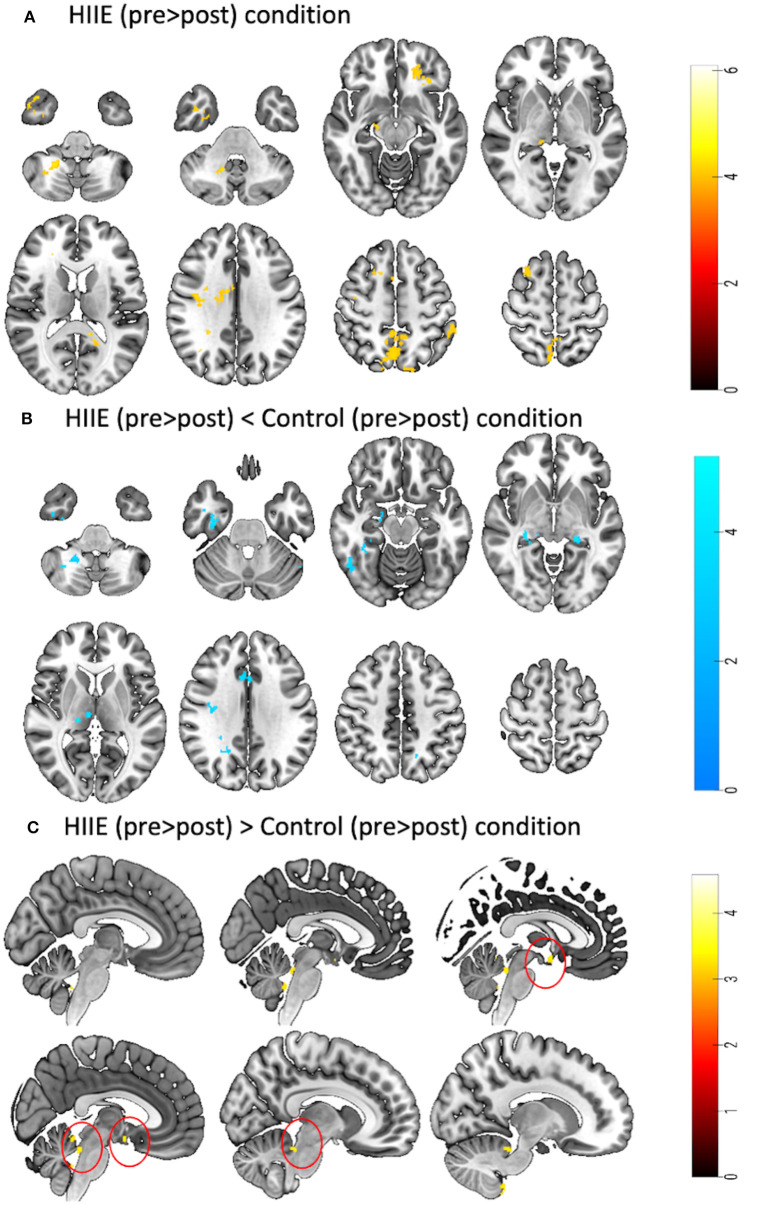
Represents a 2 × 3 repeated measures ANOVA to compare time and condition effects on zfALFF: **(A)** Results from within condition showing clusters where zfALFF was reduced after the HIIE (*p* < 0.05, k = 101), **(B)** Results from between condition showing clusters where zfALFF was reduced after the HIIE condition compared to control (*p* < 0.05, k ≥ 90), and **(C)** Results from between condition showing clusters where zfALFF increased after the HIIE condition compared to control (*p* < 0.05, k ≥ 148). zfALFF, normalized fractional amplitude of low frequency fluctuations; k = cluster size. The color bars represent the t-statistics of corresponding test.

When comparing the pre-to-post session changes within and between conditions using a 2 × 3 repeated measures ANOVA, we observed no significant main effects of time (pre vs. post), condition (low, high and control), or time × condition interaction effect. However, exploratory *post hoc* comparisons revealed significant decreases in zfALFF after the HIIE intervention (*p* < 0.05, k ≥ 101) in several brain regions including precuneus, orbitofrontal cortex, inferior temporal gyrus, thalamus, and cerebellum. For details see Figure 3A and Table 2A. Moreover, we found decreased (*p* < 0.05, k ≥ 90) and increased (*p* < 0.05, k ≥ 148) zfALFF after the HIIE compared to the control condition. The reported regions partially overlap with the pre-post changes in the HIIE condition (including precuneus, cerebellum, hippocampus, inferior temporal gyrus, middle cingulate, and precentral gyrus), along with additional regions, including thalamus, middle temporal gyrus, fusiform/parahippocampal gyrus, and frontal operculum (see Figure 3B, Table 2B). Conversely, zfALFF increases were found in hypothalamus, periaqueductal gray as well as in the pituitary (see Figure 3C, Table 2C). We did not observe any significant time effect within LIIE and control conditions, as well as between conditions (LIIE vs. control, and vice versa as well as HIIE vs. LIIE and vice versa).

**Table 2:** Significant clusters obtained from different *post-hoc* contrasts of 2 × 3 repeated measures ANOVA.

**No**.	**Cluster label**	**Side**	**Cluster size**	**MNI coordinates (mm)**			***t-*value**	***p*-value**
			**(mm3)**	**X**	**Y**	**Z**		
**A) HIIE Pre** > **Post**								
1	Cerebellum VIII	R	195	22	−51	−45	6.09	< 0.001
2	Cerebellum IX/white matter	R	101	14	−57	−33	5.61	0.025
3	Inferior temporal gyrus/Cerebellar Crus 1	L	120	−54	−57	−24	5.43	0.009
4	Precuneus/Posterior Cingulate	L	117	−12	−44	15	5.36	0.011
5	Thalamus/hippocampus	R	134	16	−24	−8	5.34	0.004
6	Precentral gyrus/white matter	R	355	40	−8	33	5.19	< 0.001
7	Superior frontal gyrus	R	205	21	18	56	5.19	< 0.001
8	Precuneus/white matter	R	106	24	−51	21	5.00	0.019
9	Middle cingulate gyrus	R	123	10	−3	32	4.91	0.008
10	Inferior orbitofrontal cortex	L	102	−21	34	−10	4.85	0.024
11	Superior frontal gyrus/white matter	R	135	20	33	24	4.84	0.004
12	Posterior orbitofrontal cortex	L	109	−27	26	−15	4.79	0.016
13	Angular gyrus	R	143	27	−42	32	4.70	0.003
14	Inferior temporal gyrus	R	281	38	3	−39	4.67	< 0.001
15	Precuneus	L	1137	−10	−64	50	4.67	< 0.001
16	Inferior parietal gyrus	L	192	−52	−42	54	4.45	< 0.001
17	Supplementary motor area	R	118	4	15	44	4.34	0.010
18	Precuneus	L	150	−8	−75	56	4.31	0.002
**B) HIIE**<**Control condition**								
1	Middle temporal gyrus/white matter	R	116	44	−3	−27	5.61	0.011
2	Precuneus	L	90	−16	−62	38	5.24	0.047
3	Thalamus	R	116	16	−24	4	5.17	0.011
4	Cerebellum VIII	R	145	22	−57	−40	5.09	0.002
5	Fusiform/Parahippocampal	R	209	36	−12	−30	4.97	< 0.001
6	Hippocampus	L	99	−22	−32	−4	4.97	0.028
7	Hippocampus	R	92	28	−30	−6	4.68	0.042
8	Precuneus/white matter	R	186	30	−45	26	4.64	< 0.001
9	Inferior temporal gyrus	R	135	51	−54	−15	4.63	0.004
10	Hippocampus	R	122	18	−24	−8	4.59	0.008
11	Middle cingulate gyrus	R	125	8	18	32	4.56	0.007
12	Precentral gyrus/white matter	R	138	36	−16	30	4.53	0.004
13	Inferior temporal gyrus/Cerebellar Crus	L	108	−52	−57	−24	4.45	0.017
14	Inferior frontal operculum	R	102	32	4	21	4.39	0.024
15	Fusiform gyrus	R	119	38	−40	−15	4.26	0.010
16	Precuneus	R	104	21	−56	33	4.17	0.021
**C) HIIE** > **Control condition**								
1	Hypothalamus/pituary	R	348	8	−6	−21	5.7	< 0.001
2	Cerebellum IX	L	301	−15	−38	−63	4.79	< 0.001
3	Periaqueductal gray	R	243	10	−38	−22	4.58	< 0.001
4	Cerebellum IX	R	396	8	−45	−70	4.24	< 0.001

HIIE, High Intensity Interval Exercise.

#### Associations between fALFF changes and PANAS

Follow-up regression analyses examined potential associations between the observed fALFF changes corresponding pre-to-post changes in the affective questionnaires. No significant associations were observed.

#### Spatial correlations of fALFF changes with neurotransmitter maps

In the exploratory whole-brain analysis for 14 neurotransmitter systems, no significant spatial correlations were observed between the pairwise differences in zfALFF and any of the neurotransmitter maps in the LIIE and the control condition. For the HIIE condition, only a marginal effect for the endocannabinoid map emerged (Fisher's z = −0.12, exact *p* = 0.029, Supplementary Figure S2), but this did not survive multiple comparison correction.

**Figure 4:**
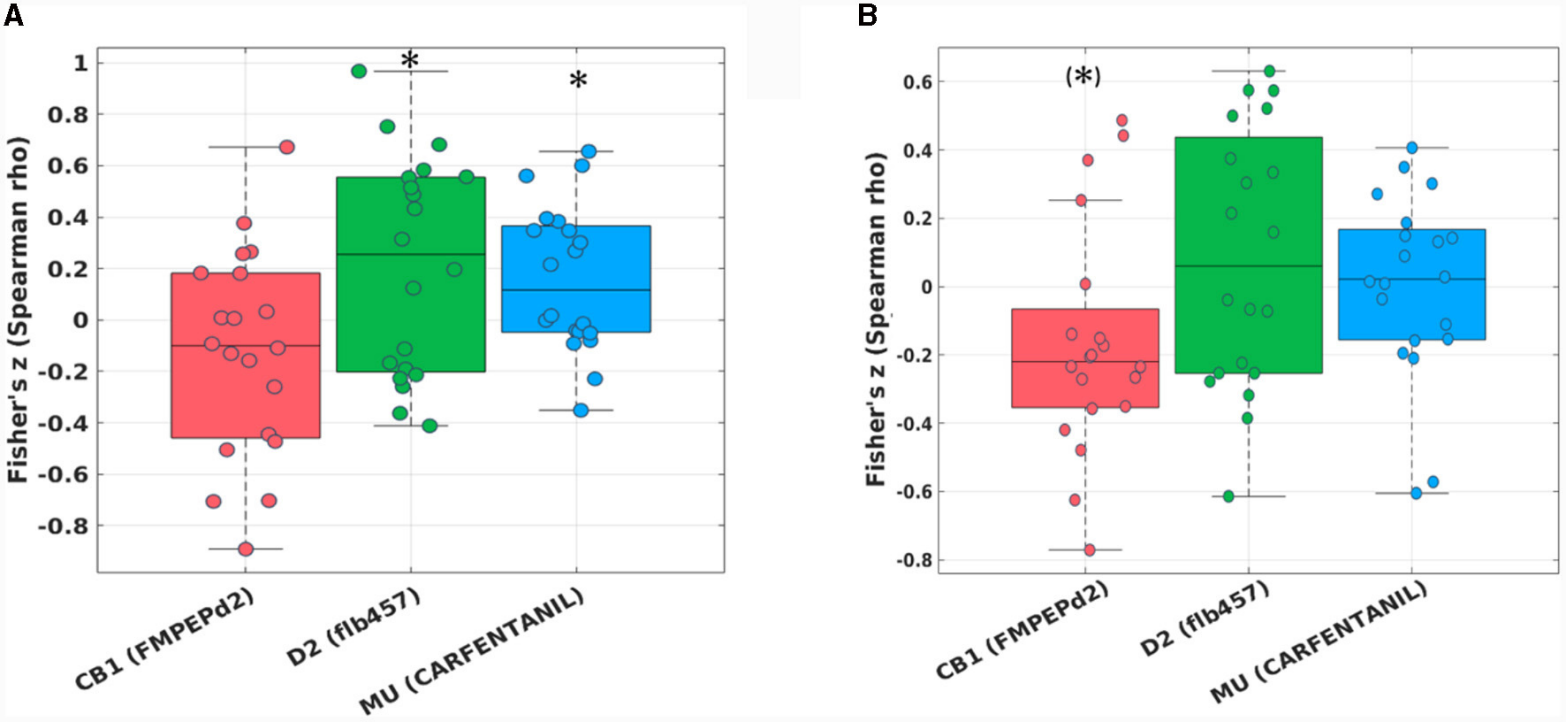
Fisher's z distribution of the Spearman correlation between the fALFF changes from pre to post in the HIIE condition and neurotransmitters (included in JuSpace toolbox) **(A)** within the “reward network,” and **(B)** within the “emotion network” in trained athletes. *significant; (*)trend.

The hypothesis-driven analysis focusing on the “reward network” showed a significant spatial correlation of the pairwise zfALFF differences and the Mu-opiodergic receptor distribution (Fisher's z = 0.16, *p* = 0.027, corrected) after HIIE (Figure 4A). Additionally, we observed a significant effect (Fisher's z = 0.22, *p* = 0.037, corrected) with the dopamine (D2) receptor distribution after the HIIE condition (Figure 4A). Again, no significant correlation was observed within the reward network at the LIIE condition or the control condition.

The hypothesis-driven analysis focusing on the “emotion network” revealed a trend spatial correlation (Fisher's z = −0.16, *p* = 0.064, corrected) between the pairwise zfALFF differences and the CB1 receptor distribution after the HIIE condition (Figure 4B).

#### fALFF-neurotransmitters correlations with mood changes

No significant correlations were observed between the changes in affective scores and the changes in spatial correlations between fALFF and the investigated neurotransmitter maps.

## Discussion

This study examined acute exercise-induced changes in resting-state zfALFF and their spatial cross-correlations with representative PET neurotransmitter distribution maps using the JuSpace toolbox (Dukart et al., 2021) to make indirect inferences about exercise-related acute neurotransmission changes. The within-subject design with three acute training sessions at different activity levels (Control, LIEE, HIIE) in a group of 20 highly trained athletes allowed determining dose-dependent influences of training intensity on resting-state zfALFF changes and their relationship with respective neurotransmitter distributions in the human brain. While both exercise conditions showed the expected positive mood effects (PANAS Positive Affect scale), no robust exercise related changes of resting-state activation levels could be observed, although exploratory analyses revealed post-acute effects in the HIIE condition. Associated with these activity changes, results of spatial cross-correlations with representative PET neurotransmitter distribution maps suggest potential involvement of opioidergic (via mu-opioid receptors) and dopaminergic (via D2 receptors) neurotransmission after HIIE, with weak evidence for an additional involvement of the endocannabinoid system (via CB1 receptors).

Exercise had the anticipated mood-improving impact, as evidenced by the higher PANAS Positive Affect following LIIE and HIIE bouts (Reed and Ones, 2006; Schmitt et al., 2019, 2020). Meanwhile, no significant increases of PANAS Negative Affect were observed for LIIE and HIIE, indicating that these exercise bouts did not induce post-acute aversive mood states. Indeed, previous evidence suggests that HIIE bouts may induce negative affect during performance (if homeostasis is disrupted to a significant extent) that is followed by a positive affective rebound immediately after exercise (Dierkes et al., 2021). The fact that PANAS Negative Affect only decreased significantly in the control condition is unexpected, given that the participants generally scored in the low range. We can only speculate whether this indicates the athletes' relieve from passively sitting on the bicycle for an extended time period.

To the best of our knowledge, zfALFF changes have not yet been studied in acute exercise intervention settings, as reported here. We did not find significant condition-by-time interaction effects, i.e., no robust proof for differential zfALFF changes in the control relative to (or between) the exercise conditions. Exploratory *post-hoc* analyses revealed preliminary evidence for zfALFF changes after the HIIE condition: Decreases in zfALFF (pre- > post-intervention) were mainly found in precuneus, orbitofrontal cortex, thalamus, and cerebellum. Compared to the control condition, HIIE was associated with differential zfALFF decreases in precuneus, temporo-occipital, midcingulate and frontal regions, thalamus, and cerebellum, whereas differential zfALFF increases were identified in hypothalamus, pituitary, and periaqueductal gray. Comparing these findings with studies using related neuroimaging techniques, we did not replicate previous ASL studies that point toward post-exercise CBF increases in the hippocampus (Steventon et al., 2020) and posterior insula (Thackray et al., 2023). Yet, the available literature is not consistent, as there were also observations of hippocampal (MacIntosh et al., 2014; Alfini et al., 2020) and mesial orbitofrontal CBF decreases (Thackray et al., 2023), the latter being in line with the present result patterns, and also with previous models that suggested transient frontal activity reductions, at least during acute exercise (Dietrich and Audiffren, 2011). The significant clusters also show limited overlap with previous rs-fMRI studies which found increased FC in sensorimotor networks (Rajab et al., 2014), or affect-reward, hippocampal, cingulo-opercular, and executive control networks (Steventon et al., 2017), although again, conflicting FC reductions were also observed (Alfini et al., 2020). Partially consistent with our earlier FC analyses (Schmitt et al., 2019, 2020), results patterns suggested intensity-dependent effects, although in the present case, only the HIIE (not the LIIE) condition showed significant differences from the control condition, and there was no significant difference between the exercise conditions. Limited statistical power may have disguised weaker exercise-related changes in the LIIE condition. At least, the result pattern argues against a curvilinear, inverted U-shape relationship with exercise intensity (as discussed in other contexts, e.g., Reed and Ones, 2006; Raichlen et al., 2013). The lack of spatial consistency between our zALFF and previous FC analyses of rs-fMRI data may relate to the fact that the FC methods capture the functional integration between brain regions, and only allow indirect assumptions about the underlying levels. In general, it should be noted that the available neuroimaging studies show substantial methodological variations, e.g., regarding age and fitness level of the participants, duration and intensity of the acute exercise, which may to some degree explain the variable or even contradictory observations. In fact, the relatively intense, but short exercise bouts during HIIE may trigger somewhat different adaptational processes than continuous trainings used in earlier studies.

Interestingly, zfALFF increases were found after HIIE in regions belonging to the hypothalamic-pituitary-adrenal (HPA) axis. One previous study examined hypothalamic CBF, and found no exercise-related effects, but using a continuous, moderate intensity training instead of HIIE (Thackray et al., 2023). The hypothalamus integrates signals from other brain nuclei as well as environmental, hormonal, metabolic, and neuronal signals from the periphery (Taouis, 2016). Thereby, vital functions such as energy homeostasis, water balance, and stress are controlled (Taouis, 2016), which are essential for adequate responses to exercise, both acutely and chronically (Cano Sokoloff and Misra, 2016). For example, in order to appropriately respond to the rise in body temperature during strenuous exercise, effector responses are triggered by the hypothalamus (Gleeson, 1998). Differential zfALFF changes also occurred in the PAG, which is an integral hub region of the opioidergic descending antinociceptive system (Bagley and Ingram, 2020). Given that pain is a prominent symptom in exercise of high intensity and/or duration it is well conceivable that exercise triggers antinociceptive responses via the PAG (Scheef et al., 2012). Beyond its role in antinociception, the PAG also fulfills many requirements of a command center for the control of breathing during exercise (Paterson, 2014). It has functional connections to higher brain areas, receives sensory input from contracting muscles, and sends efferent information to brainstem nuclei involved in cardiorespiratory control (Paterson, 2014). In conclusion, the observed zfALFF increases may reflect enhanced regulation of neurohumoral processes, as an adaption to the acute stress during the exercise bouts (see also below).

A particular innovative aspect of the current study is the parallel examination of multiple neurotransmitter effects using rsfMRI after acute exercise bouts with varying intensity. Although the JuSpace approach (Dukart et al., 2021) is *per se* indirect (i.e., spatial cross-correlations of zfALFF changes with representative neurotransmitter maps) and does not allow quantification of endogenous transmitter release (as in PET displacement studies), this MRI-based approach has the unique advantage that several neurotransmitter systems can be tested at the same time in parallel, thereby providing novel insights that help generating hypotheses for future, more transmitter-specific studies. The present results were consistent with the *a priori* proposed roles of the dopaminergic, opioidergic and, less clearly, endocannabinoid neurotransmitter systems in high-intensity exercise, as suggested by previous research (Raichlen et al., 2013; Hiura et al., 2017; Saanijoki et al., 2018a).

At whole-brain level, unconstrained analyses found no robust evidence for pre-post changes for the 14 available neurotransmitter maps after correcting for multiple comparisons, even though there was marginal evidence for the CB1 receptor map at uncorrected *p*-values, which were moreover restricted to the high-intensity condition. Even in the constrained analysis for a priori-defined emotion-related networks, this spatial correlation only approached significance. While it was shown in humans that exercise increases circulating endocannabinoid levels in an intensity-dependent manner (Raichlen et al., 2013; Saanijoki et al., 2018b), the current observation must be interpreted with caution. Here, it should also be noted that the overall spatial correlation between the zfALFF changes and the CB1 map was negative, suggesting a trend for activity decreases across endocannabinoid-rich regions: While CB1 receptors are located presynaptically, and their activation by endocannabinoids generally inhibits the release of other transmitters from these synapses (Mechoulam and Parker, 2013), CB1 receptors are expressed on both excitatory and inhibitory synapses which can have complex net effects on signal transmission at system level. Ultimately, direct proof for exercise-induced changes in brain endocannabinoid transmission will depend on future ligand PET studies, although it would be especially interesting here to examine their relationship with concomitant zfALFF (and other rs-fMRI parameter) changes in combined PET/MRI studies.

The analysis restricted to the “reward network” revealed significant spatial correlations of zFALFF changes with the dopaminergic and μ-opiodergic transmitter maps after the high-intensity exercise condition, confirming our *a priori* hypotheses. For the dopaminergic system, results showed a positive association, suggesting higher brain activity (as indicated by zfALFF) in the D2-rich regions of the reward network. This positive correlation parallels findings from the original JuSpace study, which validated the toolbox with data from an acute risperidone challenge study (Dukart et al., 2021). Human evidence on dopamine transmission changes after exercise generally remains scarce. There are earlier observations of increased peripheral dopamine metabolite levels after exercise (Marques et al., 2021), but they provide no insight into central dopamine transmission. This is currently only possible with molecular imaging methods. An early PET study already demonstrated that a gait challenge significantly increased endogenous dopaminergic release in Parkinson patients, but did not examine possible relationships with motivational behavior (Ouchi et al., 2001), which is also a limitation of the present study. While a recent study in healthy individuals reported striatal dopamine release during acute exercise that also correlated with speeded reaction times in a cognitive task (Ando et al., 2024), this does not necessarily reflect motivational changes. Another PET ligand displacement study in Parkinson patients reported that ventral striatal dopamine release after steady-state cycling was more pronounced in habitual exercisers than in sedentary subjects with Parkinson's disease (Sacheli et al., 2018), suggesting an influence of training status. Interestingly, the patient groups also showed differential ventral striatal brain activation during an fMRI reward paradigm. While not acquired in the context of the exercise session, this sort of fMRI paradigm may be an interesting addition to generate more specific information about motivational changes. In fact, an earlier study observed blunted ventral striatal responses during monetary reward anticipation and receipt after treadmill exercise, which may reflect higher levels of baseline brain activity immediately after exercise bouts (Bothe et al., 2013): this would concur with our observation, assuming that the increased spatial correlation with the D2 map within the reward network reflects higher brain activity.

There was also a positive spatial correlation between the zfALFF and the mu-opioid distribution map within the reward network regions. Both neurotransmitter systems are assumed to play complementary roles in reward processing, with dopamine being more closely related to motivational-energetic (“wanting”) aspects, and opioids being more closely related to hedonic-consummatory aspects (“liking”) of reward (Berridge and Robinson, 2003). Indeed, joint opioidergic and dopaminergic neurotransmission was detected in the ventral striatum of rats: A rostro-dorsally located “opioid hedonic hotspot” in the nucleus accumbens, mediating behavioral “liking” reactions via endorphinergic transmission (Castro and Berridge, 2014). This “liking” hotspot could be distinguished from a separate, more caudally located hotspot for “wanting” behavior mediated via dopaminergic transmission (Castro and Berridge, 2014). Interestingly, one human study observed that the individual strength of opioid release after moderate exercise training correlated positively with the brain response of reward network areas (including ventral striatum) while viewing palatable, as compared to non-palatable food pictures, which would be consistent with an opioidergic mechanisms to hedonic aspects of reward processing (Saanijoki et al., 2018b). Although we do not have behavioral results to distinguish the representations of “liking” and “wanting” in the ventral striatum, it is noteworthy that our analyses indicate a common representation of these two neurotransmitter systems and an effect of HIIE on both. This finding is also consistent with observations that central opioid release appears to be dependent on the intensity of exercise (Hiura et al., 2017; Saanijoki et al., 2018a). Previous work from our group identified exercise-induced opioidergic transmitter release, as measured with 6-O-(2-[18F]fluoroethyl)-6-O-desmethyldiprenor-phine ([18F]DPN) PET (Boecker et al., 2008). Importantly, [18F]DPN binding changes correlated with VAS euphoria change scores (Boecker et al., 2008). Why such a relationship was not found with the current indirect analysis approach remains a matter of speculation and should be addressed in future studies. Another study reported that increased opioid release was correlated with increased euphoria after a moderate intensity continuous exercise, but increased negative affect after high-intensity interval training (Saanijoki et al., 2018a), which may indicate that at very high levels of exercise strain the stress-reducing effects of opioidergic neurotransmission become more important. In this context it is interesting to revisit an abovementioned observation from the present study: It is intriguing that zfALFF changes were found in the hypothalamus and the PAG, both of which are known as core regions of the central opioidergic neurotransmitter system, but not covered by the JuSpace maps. The hypothalamus is the region where β-endorphins are produced and released, in particular in the pro-opio-melano-cortin (POMC) neurons located in the arcuate hypothalamic nucleus (ARH) and, to a lesser extent, in the nucleus of the solitary tract (Veening et al., 2012). B-endorphins from the hypothalamic POMC neurons in the ARH are released inside the central nervous system, notably to limbic structures, hypothalamic and thalamic sites, and several brainstem nuclei (Veening et al., 2012). On the other hand, β-endorphins produced in the nucleus solitarius project axons to the spinal cord. In addition to these central acting β-endorphins, those from the pituitary are released into the peripheral systemic circulation (Veening et al., 2012). Activation of the hypothalamic-pituitary-adrenal (HPA) axis by physical exercise has been demonstrated by increased endorphin levels in the pituitary (Tendzegolskis et al., 1991; Goldfarb and Jamurtas, 1997) and elevated plasma immunoreactive β-endorphin/β-lipotropin levels (Kelso et al., 1984; Mastorakos et al., 2005). Differential zfALFF changes in the PAG are equally interesting as the region is a crucial hub region of the descending antinociceptive system which is opoidergically mediated and modulated by exercise, as shown by our group in human athletes after strenuous exercise (Scheef et al., 2012). In line with our observations, it was demonstrated in mice after exposure to forced walking stress that β-endorphin levels are increased in PAG and/or medial basal hypothalamus and may be involved in stress-induced analgesia (Nakagawasai et al., 1999). Meanwhile, extensive expression of CB1 and CB2 receptors of the endocannabinoid system has been found in limbic regions and also in the hypothalamus (Ferber et al., 2021). Furthermore, CB1 receptor signaling has been shown to have an effect on the activation of the HPA axis (Hillard et al., 2016). One thus needs to consider that there are mutual interactions between the endocannabinoid systems and the opioidergic (Crombie et al., 2018; Wenzel and Cheer, 2018) and dopaminergic (Wenzel and Cheer, 2018) systems in the human brain, with indications that the opioid system is involved in the increase of endocannabinoids following exercise (Crombie et al., 2018). Yet, with the currently available technical approaches, these complex interrelationships are difficult to disentangle in humans.

In general, both ligand PET studies of exercise-induced neurotransmission changes and the proposed JuSpace-based fMRI approach only provide correlational data by design, and even if these measures show associations with the subjective and behavioral effects of acute exercise, they do not allow inferences about causal relationships. Here, selective modulation of specific transmitter functions by means of pharmacological blocking or enhancement could provide important complementary insights. Meanwhile, the available literature is small (Marques et al., 2021; Hirschbeck et al., 2022), and gives few specific insights with regard to emotional/motivational changes after exercise (e.g., Siebers et al., 2021). Ideally, both methodological approaches would be combined, although this affords even more repeated scanning, which is probably more feasible with fMRI (due to radiation exposure regulations for PET). At least, imaging studies may provide a useful starting point for the selection of pharmacological manipulations (where JuSpace would allow to screen multiple transmitter systems in parallel).

This study is not without limitations: We constrained our analyses of the “emotion network” and the “reward network” to three transmitter systems described in the context of affect and reward processing. The reported effects were only detectable in the high-intensity condition and effect sizes were too weak to survive corrections for multiple testing of all available PET transmitter maps. Therefore, future studies of this kind should attempt to measure larger samples in order to detect further neurotransmitter involvement related to exercise. Moreover, future examinations should enroll all sexes, which was not the case in this pilot study. While we decided to examine cycling as a common form of training, we cannot make direct inferences for other types of endurance exercise, e.g., running: even if general fitness levels are comparable, varying demands of the exercise modes (e.g., regarding active muscle mass, motor unit recruitment patterns) can alter physiological responses like oxygen uptake (Kilding and Jones, 2008). Accordingly, generalizability should be scrutinized by examining additional (or mixed) athlete groups. We can only speculate on the association with neurotransmitter effects in those regions like the hypothalamus and the PAG which were not covered by the PET tracer distribution maps. Finally, we must acknowledge that although the study design controlled for the influence of examination time by keeping it the same for each participant throughout the study (i.e., reducing the influence of circadian rhythms as a confounding factor at the individual level), examination took place during normal working hours (08:00 to 17:00), and the sample size did not allow for a systematic analysis of possible moderator effects of the time of day.

In summary, this is the first attempt to study exercise-induced neurotransmission non-invasively and in a rather holistic manner using rs-fMRI in humans. Our approach of studying zfALFF changes in the context of acute exercise bouts differing in intensity has provided novel information on the neurobiology of exercise, extending previous evidence on the particular relevance of the opioidergic, the dopaminergic and the endocannabinoid systems in high-intensity physical activity. Interactions between these transmitter systems have been described and should be studied further *in vivo*, e.g., between endocannabinoid signaling and endorphin (Parolaro et al., 2010; Wenzel and Cheer, 2018) or dopamine release (Covey et al., 2017; Wenzel and Cheer, 2018), in the specific context of exercise physiology. Yet, this necessitates future studies in larger samples which also provide more statistical power to discover additional relationships for other neurotransmitter systems, and also to detect correlations with the behavioral effects of acute exercise. A deeper understanding of the neuropharmacological underpinning of acute exercise effects is not only interesting from the perspective of basic science, but may ultimately also help to further consolidate the role of exercise trainings in clinical populations with dysregulated transmitter function.

## Data availability statement

The datasets presented in this article are not readily available because volunteers will need to provide consent possibly restricting deliverable data sets. Requests to access the datasets should be directed to henning.boecker@ukbonn.de.

## Ethics statement

The studies involving humans were approved by Ethics Committee at the Medical Faculty of the Rheinische Friedrich-Wilhelms-Universität Bonn. The studies were conducted in accordance with the local legislation and institutional requirements. The participants provided their written informed consent to participate in this study.

## Author contributions

HB: Conceptualization, Investigation, Resources, Supervision, Validation, Writing—original draft, Writing—review & editing. MD: Conceptualization, Methodology, Supervision, Visualization, Writing—review & editing. AM: Conceptualization, Data curation, Formal analysis, Investigation, Methodology, Project administration, Supervision, Validation, Writing—original draft, Writing—review & editing. LB: Data curation, Formal analysis, Investigation, Writing—original draft, Writing—review & editing. JW: Data curation, Investigation, Writing—review & editing. ML: Data curation, Investigation, Writing—review & editing. CM: Data curation, Formal analysis, Investigation, Methodology, Resources, Supervision, Writing—review & editing. UM: Data curation, Investigation, Resources, Writing—review & editing. AR: Funding acquisition, Resources, Writing—review & editing. UA: Funding acquisition, Resources, Writing—review & editing. JD: Methodology, Software, Supervision, Writing—review & editing. NU: Conceptualization, Data curation, Formal analysis, Investigation, Methodology, Software, Validation, Writing—original draft, Writing—review & editing.

## References

[B1] Aghourian M. Legault-Denis C. Soucy J.-.P Rosa-Neto P. Gauthier S.. (2017). Quantification of brain cholinergic denervation in Alzheimer's disease using PET imaging with [(18)F]-FEOBV. Mol. Psychiat. 22, 1531–1538. 10.1038/mp.2017.183 28894304

[B2] Aiello M. Salvatore E. Cachia A. Pappatà S. Cavaliere C. Prinster A.. (2015). Relationship between simultaneously acquired resting-state regional cerebral glucose metabolism and functional MRI: a PET/MR hybrid scanner study. Neuroimage 113, 111–121. 10.1016/j.neuroimage.2015.03.017 25791784

[B3] Alfini A. J. Won J. Weiss L. R. Nyhuis C. C. Shackman A. J. Spira A. P.. (2020). Impact of exercise on older adults' mood is moderated by sleep and mediated by altered brain connectivity. Soc. Cogn. Affect. Neurosci. 15, 1238–1251. 10.1093/scan/nsaa149 33201227 PMC7745152

[B4] Allen M. Thierman J. Hamilton D. (1983). Naloxone eye drops reverse the miosis in runners–implications for an endogenous opiate test. Can. J. Appl. Sport Sci. 8, 98–103. 10.1249/00005768-198315020-00295 6883621

[B5] Ando S. Fujimoto T. Sudo M. Watanuki S. Hiraoka K. Takeda K.. (2024). The neuromodulatory role of dopamine in improved reaction time by acute cardiovascular exercise. J. Physiol. 602, 461–484. 10.1113/JP285173 38165254

[B6] Bagley E. E. Ingram S. L. (2020). Endogenous opioid peptides in the descending pain modulatory circuit. Neuropharmacology 173:108131. 10.1016/j.neuropharm.2020.108131 32422213 PMC7313723

[B7] Basso J. C. Suzuki W. A. (2017). The effects of acute exercise on mood, cognition, neurophysiology, and neurochemical pathways: a review. Brain Plast 2, 127–152. 10.3233/BPL-160040 29765853 PMC5928534

[B8] Berridge K. C. Robinson T. E. (2003). Parsing reward. Trends Neurosci. 26, 507–513. 10.1016/S0166-2236(03)00233-9 12948663

[B9] Boecker H. Drzezga A. (2016). A perspective on the future role of brain pet imaging in exercise science. Neuroimage 131, 73–80. 10.1016/j.neuroimage.2015.10.021 26477649

[B10] Boecker H. Henriksen G. Sprenger T. Miederer I. Willoch F. Valet M.. (2008). Positron emission tomography ligand activation studies in the sports sciences: measuring neurochemistry in vivo. Methods 45, 307–318. 10.1016/j.ymeth.2008.07.003 18674621

[B11] Boecker H. Hillman C. H. Scheef L. Strüder H. K. (2012). Functional Neuroimaging in Exercise and Sport Sciences. New York, NY: Springer. 10.1007/978-1-4614-3293-7

[B12] Boecker H. Sprenger T. Spilker M. E. Henriksen G. Koppenhoefer M. Wagner K. J.. (2008). The runner's high: opioidergic mechanisms in the human brain. Cereb Cortex 18, 2523–2531. 10.1093/cercor/bhn013 18296435

[B13] Booth M. (2000). Assessment of physical activity: an international perspective. Res. Q Exerc. Sport 71, 114–120. 10.1080/02701367.2000.11082794 25680021

[B14] Borg G. (1998). Borg's Perceived Exertion and Pain Scales. London: Human Kinetics.

[B15] Bothe N. Zschucke E. Dimeo F. Heinz A. Wüstenberg T. Ströhle A.. (2013). Acute exercise influences reward processing in highly trained and untrained men. Med. Sci. Sports Exerc. 45, 583–591. 10.1249/MSS.0b013e318275306f 23059859

[B16] Cano Sokoloff N. Misra M. (2016). Ackerman KExercise E, training, and the hypothalamic-pituitary-gonadal axis in men and women. Front. Horm. Res. 47, 27–43. 10.1159/000445154 27348623 PMC7043068

[B17] Castro D. C. Berridge K. C. (2014). Opioid hedonic hotspot in nucleus accumbens shell: mu, delta, and kappa maps for enhancement of sweetness “liking” and “wanting”. J. Neurosci. 34, 4239–4250. 10.1523/JNEUROSCI.4458-13.2014 24647944 PMC3960467

[B18] Covey D. P. Mateo Y. Sulzer D. Cheer J. F. (2017). Endocannabinoid modulation of dopamine neurotransmission. Neuropharmacology 124, 52–61. 10.1016/j.neuropharm.2017.04.033 28450060 PMC5608040

[B19] Crombie K. M. Brellenthin A. G. Hillard C. J. Koltyn K. F. (2018). Endocannabinoid and opioid system interactions in exercise-induced hypoalgesia. Pain Med. 19, 118–123. 10.1093/pm/pnx058 28387833 PMC6454785

[B20] De Pauw K. Roelands B. Cheung S. S. de Geus B. Rietjens G. Meeusen R.. (2013). Guidelines to classify subject groups in sport-science research. Int. J. Sports Physiol. Perform 8, 111–122. 10.1123/ijspp.8.2.111 23428482

[B21] Dera A. M. Shen T. Thackray A. E. Hinton E. C. King J. A. James L.. (2023). The influence of physical activity on neural responses to visual food cues in humans: a systematic review of functional magnetic resonance imaging studies. Neurosci. Biobehav. Rev. 152:105247. 10.1016/j.neubiorev.2023.105247 37236384

[B22] Desai S. Borg B. Cuttler C. Crombie K. M. Rabinak C. A. Hill M. N.. (2022). A systematic review and meta-analysis on the effects of exercise on the endocannabinoid system. Cannabis Cannab. Res. 7, 388–408. 10.1089/can.2021.0113 34870469 PMC9418357

[B23] Dierkes K. Maturana F. M. Rösel I. Martus P. Nie,ß A. M. Thiel A.. (2021). Different endurance exercise modalities, different affective response: a within-subject study. Front. Psychol. 12:686661. 10.3389/fpsyg.2021.686661 34484040 PMC8411706

[B24] Dietrich A. Audiffren M. (2011). The reticular-activating hypofrontality (RAH) model of acute exercise. Neurosci. Biobehav. Rev. 35, 1305–1325. 10.1016/j.neubiorev.2011.02.001 21315758

[B25] Dorling J. Broom D. R. Burns S. F. Clayton D. J. Deighton K. James L. J.. (2018). Acute and chronic effects of exercise on appetite, energy intake, and appetite-related hormones: the modulating effect of adiposity, sex, and habitual physical activity. Nutrients 10:1140. 10.3390/nu10091140 30131457 PMC6164815

[B26] Dukart J. Holiga Š. Chatham C. Hawkins P. Forsyth A. McMillan R.. (2018). Cerebral blood flow predicts differential neurotransmitter activity. Sci. Rep. 8:4074. 10.1038/s41598-018-22444-0 29511260 PMC5840131

[B27] Dukart J. Holiga S. Rullmann M. Lanzenberger R. Hawkins P. C. T. Mehta M. A.. (2021). JuSpace: A tool for spatial correlation analyses of magnetic resonance imaging data with nuclear imaging derived neurotransmitter maps. Hum. Brain Mapp. 42, 555–566. 10.1002/hbm.25244 33079453 PMC7814756

[B28] Esteban O. Birman D. Schaer M. Koyejo O. O. Poldrack R. A. Gorgolewski K. J. (2017). MRIQC: advancing the automatic prediction of image quality in MRI from unseen sites. PLoS ONE 12:e0184661. 10.1371/journal.pone.0184661 28945803 PMC5612458

[B29] Esteban O. Markiewicz C. J. Blair R. W. Moodie C. A. Isik A. I. Erramuzpe A.. (2019). fMRIPrep: a robust preprocessing pipeline for functional MRI. Nat. Methods 16, 111–116. 10.1038/s41592-018-0235-4 30532080 PMC6319393

[B30] Ferber S. G. Hazani R. Shoval G. Weller A. (2021). Targeting the endocannabinoid system in borderline personality disorder: corticolimbic and hypothalamic perspectives. Curr. Neuropharmacol. 19, 360–371. 10.2174/1570159X18666200429234430 32351183 PMC8033970

[B31] Fonov V. Evans A. C. Botteron K. Almli C. R. McKinstry R. C. Collins D. L.. (2011). Unbiased average age-appropriate atlases for pediatric studies. NeuroImage 54, 313–327. 10.1016/j.neuroimage.2010.07.033 20656036 PMC2962759

[B32] Forteza F. Giorgini G. Raymond F. (2021). Neurobiological processes induced by aerobic exercise through the endocannabinoidome. Cells 10:938. 10.3390/cells10040938 33920695 PMC8072750

[B33] Galdino G. Romero T. da Silva J. F. P. Aguiar D. de Paula A. M. Cruz J.. (2014). Acute resistance exercise induces antinociception by activation of the endocannabinoid system in rats. Anesth. Analg. 119, 702–715. 10.1213/ANE.0000000000000340 24977916 PMC4139418

[B34] Gamelin F.-. X Aucouturier J. Iannotti F. A. Piscitelli F. Mazzarella E.. (2016). Exercise training and high-fat diet elicit endocannabinoid system modifications in the rat hypothalamus and hippocampus. J. Physiol. Biochem. 73, 335–347. 10.1007/s13105-017-0557-1 28283967

[B35] García-Gómez F. J. García-Solís D. Luis-Simón F. J. Marín-Oyaga V. A. Carrillo F. Mir P.. (2018). Elaboración de una plantilla de SPM para la normalización de imágenes de PET con 18F-DOPA. Imagen Diagnóst. 9, 23–25. 10.33588/imagendiagnostica.901.2 23570700

[B36] Gleeson M. (1998). Temperature regulation during exercise. Int. J. Sports Med. 19, S96–S99. 10.1055/s-2007-971967 9694408

[B37] Goldfarb A. H. Jamurtas A. Z. (1997). Beta-endorphin response to exercise: an update. Sports Med. 24, 8–16. 10.2165/00007256-199724010-00002 9257407

[B38] Gorrell S. Shott M. E. Frank G. K. W. (2022). Associations between aerobic exercise and dopamine-related reward-processing: Informing a model of human exercise engagement. Biol. Psychol. 171:108350. 10.1016/j.biopsycho.2022.108350 35561818 PMC9869713

[B39] Guszkowska M. (2004). Effects of exercise on anxiety, depression and mood. Psychiatr. Pol. 38, 611–620. 15518309

[B40] Hansen J. Y. Shafiei G. Markello R. D. Smart K. Cox S. M. L. Nørgaard M.. (2022). Mapping neurotransmitter systems to the structural and functional organization of the human neocortex. Nat. Neurosci. 25, 1569–1581. 10.1038/s41593-022-01186-3 36303070 PMC9630096

[B41] Hattori S. Naoi M. Nishino H. (1994). Striatal dopamine turnover during treadmill running in the rat: relation to the speed of running. Brain Res. Bull. 35, 41–49. 10.1016/0361-9230(94)90214-3 7953756

[B42] Hautzinger M. Bailer M. Worall H. Keller F. (1994). Beck-depressions-inventar (BDI). Bern: Huber.

[B43] Hesse S. Becker G.-.A Rullmann M. Bresch A. Luthardt J.. (2017). Central noradrenaline transporter availability in highly obese, non-depressed individuals. Eur. J. Nucl. Med. Mol. Imaging 44, 1056–1064. 10.1007/s00259-016-3590-3 28066877 PMC5538358

[B44] Hillard C. J. Beatka M. Sarvaideo J. (2016). Endocannabinoid signaling and the hypothalamic-pituitary-adrenal axis. Compr. Physiol. 7, 1–15. 10.1002/cphy.c160005 28134998 PMC5871916

[B45] Hirschbeck A. Leao D. S. Wagner E. Hasan A. Roeh A. (2022). Psychiatric medication and physical performance parameters – Are there implications for treatment? Front. Psychiat. 13:985983. 10.3389/fpsyt.2022.985983 36147967 PMC9488519

[B46] Hiura M. Sakata M. Ishii K. Toyohara J. Oda K. Nariai T.. (2017). Central mu-opioidergic system activation evoked by heavy and severe-intensity cycling exercise in humans: a pilot study using positron emission tomography with 11C-carfentanil. Int. J. Sports Med. 38, 19–26. 10.1055/s-0042-114779 28073122

[B47] Jenkinson M. Bannister P. Brady M. Smith S. (2002). Improved optimization for the robust and accurate linear registration and motion correction of brain images. NeuroImage 17, 825–841. 10.1006/nimg.2002.1132 12377157

[B48] Jenkinson M. Beckmann C. F. Behrens T. E. Woolrich M. W. Smith S. M. (2012). FSL. Neuroimage. 62, 782–790. 10.1016/j.neuroimage.2011.09.015 21979382

[B49] Kaller S. Rullmann M. Patt M. Becker G. A. Luthardt J. Girbardt J.. (2017). Test-retest measurements of dopamine D(1)-type receptors using simultaneous PET/MRI imaging. Eur. J. Nucl. Med. Mol. Imaging 44, 1025–1032. 10.1007/s00259-017-3645-0 28197685

[B50] Kantonen T. Karjalainen T. Isojärvi J. Nuutila P. Tuisku J. Rinne J.. (2020). Interindividual variability and lateralization of mu-opioid receptors in the human brain. Neuroimage 217, 116922. 10.1016/j.neuroimage.2020.116922 32407992

[B51] Kantonen T. Karjalainen T. Pekkarinen L. Isojärvi J. Kalliokoski K. Kaasinen V.. (2021). Cerebral μ-opioid and CB1 receptor systems have distinct roles in human feeding behavior. Translat. Psychiat. 11:442. 10.1038/s41398-021-01559-5 34453034 PMC8397789

[B52] Kantonen T. Pekkarinen L. Karjalainen T. Bucci M. Kalliokoski K. Haaparanta-Solin M.. (2022). Obesity risk is associated with altered cerebral glucose metabolism and decreased mu-opioid and CB(1) receptor availability. Int. J. Obes. 46, 400–407. 10.1038/s41366-021-00996-y 34728775 PMC8794779

[B53] Kelso T. B. Herbert W. G. Gwazdauskas F. C. Goss F. L. Hess J. L. (1984). Exercise-thermoregulatory stress and increased plasma beta-endorphin/beta-lipotropin in humans. J. Appl. Physiol. Respir. Environ. Exerc. Physiol. 57, 444–449. 10.1152/jappl.1984.57.2.444 6088450

[B54] Kilding A. E. Jones A. M. (2008). VO_2_ ‘overshoot' during moderate-intensity exercise in endurance-trained athletes: the influence of exercise modality. Respir. Physiol. Neurobiol. 160, 139–146. 10.1016/j.resp.2007.09.004 17981522

[B55] Klass M. Roelands B. Lévénez M. Fontenelle V. Pattyn N. Meeusen R.. (2012). Effects of noradrenaline and dopamine on supraspinal fatigue in well-trained men. Med. Sci. Sports Exerc. 44, 2299–2308. 10.1249/MSS.0b013e318265f356 22776872

[B56] Kleinert J. (2006). Adjektivliste zur erfassung der wahrgenommenen körperlichen verfassung (WKV). Zeitschr. Sportpsychol. 13, 156–164. 10.1026/1612-5010.13.4.156

[B57] Knab A. M. Lightfoot J. T. (2010). Does the difference between physically active and couch potato lie in the dopamine system? Int. J. Biol. Sci. 6, 133–150. 10.7150/ijbs.6.133 20224735 PMC2836544

[B58] Krohne H. W. Egloff B. Kohlmann C.-.W. (1996). Untersuchungen mit einer deutschen Version der “Positive and Negative Affect Schedule” (PANAS) [Investigations with a German version of the Positive and Negative Affect Schedule (PANAS)]. Diagnostica 42, 139–156. 10.1037/t49650-000

[B59] Landman B. Warfield S. (2012). MICCAI workshop on multi-atlas labeling. Vol. MICCAI Grand Challenge and Workshop on Multi-Atlas Labeling. Nice, France: CreateSpace Independent Publishing Platform.

[B60] Laurikainen H. Tuominen L. Tikka M. Merisaari H. Armio R. L. Sormunen E.. (2019). Sex difference in brain CB1 receptor availability in man. NeuroImage 184, 834–842. 10.1016/j.neuroimage.2018.10.013 30296558

[B61] Lewis R. G. Florio E. Punzo D. Borrelli E. (2021). The brain's reward system in health and disease. Adv. Exp. Med. Biol. 1344, 57–69. 10.1007/978-3-030-81147-1_4 34773226 PMC8992377

[B62] MacIntosh B. J. Crane D. E. Sage M. D. Rajab A. S. Donahue M. J. McIlroy W. E.. (2014). Impact of a single bout of aerobic exercise on regional brain perfusion and activation responses in healthy young adults. PLoS ONE 9:e85163. 10.1371/journal.pone.0085163 24416356 PMC3885687

[B63] Marques A. Marconcin P. Werneck A. O. Ferrari G. Gouveia É. R. Kliegel M.. (2021). Bidirectional association between physical activity and dopamine across adulthood—a systematic review. Brain Sci. 11:829. 10.3390/brainsci11070829 34201523 PMC8301978

[B64] Mastorakos G. Pavlatou M. Diamanti-Kandarakis E. Chrousos G. P. (2005). Exercise and the stress system. Hormones 4, 73–89. 16613809

[B65] Matei D. Trofin D. Iordan D. A. Onu I. Condurache I. Ionite C.. (2023). The endocannabinoid system and physical exercise. Int. J. Mol. Sci. 24:1989. 10.3390/ijms24031989 36768332 PMC9916354

[B66] Maurer A. Klein J. Claus J. Upadhyay N. Henschel L. Martin J. A.. (2022). Effects of a 6-month aerobic exercise intervention on mood and amygdala functional plasticity in young untrained subjects. Int. J. Environ. Res. Public Health 19:6078. 10.3390/ijerph19106078 35627616 PMC9140773

[B67] Mechoulam R. Parker L. A. (2013). The endocannabinoid system and the brain. Annu. Rev. Psychol. 64, 21–47. 10.1146/annurev-psych-113011-143739 22804774

[B68] Meeusen R. (2010). Roelands Central fatigue B and neurotransmitters, can thermoregulation be manipulated? Scand J. Med. Sci. Sports 20, 19–28. 10.1111/j.1600-0838.2010.01205.x 21029187

[B69] Meeusen R. DeMeirleir K. (1995). Exercise and brain neurotransmission. Sports Med. 20, 160–188. 10.2165/00007256-199520030-00004 8571000

[B70] Meeusen R. Piacentini M. F. Meirleir D.e. (2001). K. Brain microdialysis in exercise research. Sports Med. 31, 965–983. 10.2165/00007256-200131140-00002 11735681

[B71] Mulser L. Moreau D. (2023). Effect of acute cardiovascular exercise on cerebral blood flow: a systematic review. Brain Res. 1809:148355. 10.1016/j.brainres.2023.148355 37003561

[B72] Nakagawasai O. Tadano T. No K. T. Niijima F. Sakurada S. Endo Y.. (1999). Changes in beta-endorphin and stress-induced analgesia in mice after exposure to forced walking stress. Methods Find Exp. Clin. Pharmacol. 21, 471–476. 10.1358/mf.1999.21.7.550109 10544390

[B73] Norris C. J. Gollan J. Berntson G. G. Cacioppo J. T. (2010). The current status of research on the structure of evaluative space. Biol. Psychol. 84, 422–436. 10.1016/j.biopsycho.2010.03.011 20346389 PMC2894997

[B74] Oldfield R. C. (1971). The assessment and analysis of handedness: the Edinburgh inventory. Neuropsychologia 9, 97–113. 10.1016/0028-3932(71)90067-4 5146491

[B75] Ouchi Y. Kanno T. Okada H. Yoshikawa E. Futatsubashi M. Nobezawa S.. (2001). Changes in dopamine availability in the nigrostriatal and mesocortical dopaminergic systems by gait in Parkinson's disease. Brain 124, 784–92. 10.1093/brain/124.4.784 11287377

[B76] Parkes L. Fulcher B. Yücel M. Fornito A. (2018). An evaluation of the efficacy, reliability, and sensitivity of motion correction strategies for resting-state functional MRI. Neuroimage 171, 415–436. 10.1016/j.neuroimage.2017.12.073 29278773

[B77] Parolaro D. Rubino T. Viganò D. Massi P. Guidali C. Realini N.. (2010). Cellular mechanisms underlying the interaction between cannabinoid and opioid system. Curr. Drug Targets 11, 393–405. 10.2174/138945010790980367 20017730

[B78] Paterson D. J. (2014). Defining the neurocircuitry of exercise hyperpnoea. J. Physiol. 592, 433–444. 10.1113/jphysiol.2013.261586 23918772 PMC3930430

[B79] Raichlen D. A. Foster A. D. Seillier A. Giuffrida A. Gerdeman G. L. (2013). Exercise-induced endocannabinoid signaling is modulated by intensity. Eur. J. Appl. Physiol. 113, 869–875. 10.1007/s00421-012-2495-5 22990628

[B80] Rajab A. S. Crane D. E. Middleton L. E. Robertson A. D. Hampson M. MacIntosh B. J.. (2014). A single session of exercise increases connectivity in sensorimotor-related brain networks: a resting-state fMRI study in young healthy adults. Front. Hum. Neurosci. 8:625. 10.3389/fnhum.2014.00625 25177284 PMC4132485

[B81] Reed J. Ones D. S. (2006). The effect of acute aerobic exercise on positive activated affect: a meta-analysis. Psychol. Sport Exer. 7, 477–514. 10.1016/j.psychsport.2005.11.003

[B82] Robison L. S. Swenson S. Hamilton J. Thanos P. K. (2018). Exercise reduces dopamine D1R and increases D2R in rats: implications for addiction. Med. Sci. Sports Exerc. 50, 1596–1602. 10.1249/MSS.0000000000001627 29613999

[B83] Roelands B. Hasegawa H. Watson P. Piacentini M. F. Buyse L. DeSchutter G.. (2008). The effects of acute dopamine reuptake inhibition on performance. Med. Sci. Sports Exerc. 40, 879–885. 10.1249/MSS.0b013e3181659c4d 18408610

[B84] Roelands B. Meeusen R. (2010). Alterations in central fatigue by pharmacological manipulations of neurotransmitters in normal and high ambient temperature. Sports Med. 40, 229–246. 10.2165/11533670-000000000-00000 20199121

[B85] Saanijoki T. Nummenmaa L. Tuulari J. J. Tuominen L. Arponen E. Kalliokoski K. K.. (2018b). Aerobic exercise modulates anticipatory reward processing via the mu-opioid receptor system. Hum. Brain Mapp. 39, 3972–3983. 10.1002/hbm.24224 29885086 PMC6866313

[B86] Saanijoki T. Tuominen L. Tuulari J. J. Nummenmaa L. Arponen E. Kalliokoski K.. (2018a). Opioid release after high-intensity interval training in healthy human subjects. Neuropsychopharmacology 43, 246–254. 10.1038/npp.2017.148 28722022 PMC5729560

[B87] Sacheli M. A. Murray D. K. Vafai N. Cherkasova M. V. Dinelle K. Shahinfard E.. (2018). Habitual exercisers versus sedentary subjects with Parkinson's disease: multimodal PET and fMRI study. Mov. Disord. 33, 1945–1950. 10.1002/mds.27498 30376184

[B88] Sandiego C. M. Gallezot J.-. D Lim K. Ropchan J. Lin S-f Gao H.. (2015). Reference Region Modeling Approaches for Amphetamine Challenge Studies with [11C]FLB 457 and PET. J. Cerebr. Blood Flow Metabol. 35, 623–629. 10.1038/jcbfm.2014.237 25564239 PMC4420880

[B89] Savli M. Bauer A. Mitterhauser M. Ding Y.-. S Hahn A.. (2012). Normative database of the serotonergic system in healthy subjects using multi-tracer PET. Neuroimage 63, 447–459. 10.1016/j.neuroimage.2012.07.001 22789740

[B90] Scheef L. Jankowski J. Daamen M. Weyer G. Klingenberg M. Renner J.. (2012). An fMRI study on the acute effects of exercise on pain processing in trained athletes. Pain 153, 1702–1714. 10.1016/j.pain.2012.05.008 22704853

[B91] Schmitt A. Upadhyay N. Martin J. A. Rojas S. Strüder H. K. Boecker H.. (2019). Modulation of distinct intrinsic resting state brain networks by acute exercise bouts of differing intensity. Brain Plast. 5, 39–55. 10.3233/BPL-190081 31970059 PMC6971822

[B92] Schmitt A. Upadhyay N. Martin J. A. Vega S. R. Strüder H. K. Boecker H.. (2020). Affective modulation after high-intensity exercise is associated with prolonged amygdalar-insular functional connectivity increase. Neural Plast. 2020:7905387. 10.1155/2020/7905387 32300362 PMC7132580

[B93] Schott B. H. Minuzzi L. Krebs R. M. Elmenhorst D. Lang M. Winz O. H.. (2008). Mesolimbic functional magnetic resonance imaging activations during reward anticipation correlate with reward-related ventral striatal dopamine release. J. Neurosci. 28, 14311–14319. 10.1523/JNEUROSCI.2058-08.2008 19109512 PMC6671462

[B94] Sheehan D. V. Lecrubier Y. Sheehan K. H. Amorim P. Janavs J. Weiller E.. (1998). The Mini-International Neuropsychiatric Interview (M.I.N.I.): the development and validation of a structured diagnostic psychiatric interview for DSM-IV and ICD-10. J. Clin. Psychiat. 59, 22–33. 10.1037/t18597-000 9881538

[B95] Siebers M. Biedermann S. V. Bindila L. Lutz B. Fuss J. (2021). Exercise-induced euphoria and anxiolysis do not depend on endogenous opioids in humans. Psychoneuroendocrinology 126:105173. 10.1016/j.psyneuen.2021.105173 33582575

[B96] Smart K. Cox S. M. L. Scala S. G. Tippler M. Jaworska N. Boivin M.. (2019). Sex differences in [(11)C]ABP688 binding: a positron emission tomography study of mGlu5 receptors. Eur. J. Nucl. Med. Mol. Imaging 46, 1179–1183. 10.1007/s00259-018-4252-4 30627817 PMC6451701

[B97] Spielberger C. (1983). Manual for the State-Trait Anxiety Inventory. Palo Alto, CA: Consulting Psychologists Press. 10.1037/t06496-000

[B98] Stark S. M. Kirwan C. B. Stark C. E. L. (2019). Mnemonic similarity task: a tool for assessing hippocampal integrity. Trends Cogn. Sci. 23, 938–951. 10.1016/j.tics.2019.08.003 31597601 PMC6991464

[B99] Steventon J. J. Foster C. Furby H. Helme D. Wise R. G. Murphy K.. (2017). The acute effects of aerobic exercise on the functional connectivity of human brain networks. Brain Plast. 2, 171–190. 10.3233/BPL-160039 29765855 PMC5928541

[B100] Steventon J. J. Foster C. Furby H. Helme D. Wise R. G. Murphy K. (2020). Hippocampal blood flow is increased after 20 min of moderate-intensity exercise. Cerebral Cortex 30, 525–533. 10.1093/cercor/bhz104 31216005 PMC7703728

[B101] Taouis M. (2016). MicroRNAs in the hypothalamus. Best Pract. Res. Clin. Endocrinol. Metab. 30, 641–651. 10.1016/j.beem.2016.11.006 27923457

[B102] Taylor P. A. Saad Z. S. (2013). FATCAT: (an efficient) Functional and Tractographic Connectivity Analysis Toolbox. Brain Connect 3, 523–535. 10.1089/brain.2013.0154 23980912 PMC3796333

[B103] Tendzegolskis Z. Viru A. Orlova E. (1991). Exercise-induced changes of endorphin contents in hypothalamus, hypophysis, adrenals and blood plasma. Int. J. Sports Med. 12, 495–497. 10.1055/s-2007-1024721 1752719

[B104] Thackray A. E. Hinton E. C. Alanazi T. M. Dera A. M. Fujihara K. Hamilton-Shield J. P.. (2023). Exploring the acute effects of running on cerebral blood flow and food cue reactivity in healthy young men using functional magnetic resonance imaging. Hum. Brain Mapp. 44, 3461–3938. 10.1002/hbm.26314 37145965 PMC10203797

[B105] Thayer R. E. Newman J. R. McClain T. M. (1994). Self-regulation of mood: strategies for changing a bad mood, raising energy, reducing tension. J. Pers. Soc. Psychol. 67, 910–925. 10.1037/0022-3514.67.5.910 7983582

[B106] Veening J. G. Gerrits P. O. Barendregt H. P. (2012). Volume transmission of beta-endorphin via the cerebrospinal fluid; a review. Fluids Barr. CNS 9:16. 10.1186/2045-8118-9-16 PMC3439317 22883598

[B107] Wang S. Peterson D. J. Gatenby J. C. Li W. Grabowski T. J. Madhyastha T. M.. (2017). Evaluation of field map and nonlinear registration methods for correction of susceptibility artifacts in diffusion MRI. Front. Neuroinform. 11:17. 10.3389/fninf.2017.00017 28270762 PMC5318394

[B108] Watson P. Hasegawa H. Roelands B. Piacentini M. F. Looverie R. Meeusen R.. (2005). Acute dopamine/noradrenaline reuptake inhibition enhances human exercise performance in warm, but not temperate conditions. J. Physiol. 565, 873–83. 10.1113/jphysiol.2004.079202 15831540 PMC1464564

[B109] Weaver B. Bedard M. McAuliffe J. (2013). Evaluation of a 10-minute version of the attention network test. Clin. Neuropsychol. 27, 1281–1299. 10.1080/13854046.2013.851741 24205860

[B110] Wenzel J. M. Cheer J. F. (2018). Endocannabinoid regulation of reward and reinforcement through interaction with dopamine and endogenous opioid signaling. Neuropsychopharmacology 43, 103–115. 10.1038/npp.2017.126 28653666 PMC5719091

[B111] Wollseiffen P. Ghadiri A. Scholz A. Strüder H. K. Herpers R. Peters T.. (2016). Short bouts of intensive exercise during the workday have a positive effect on neuro-cognitive performance. Stress Health 32, 514–523. 10.1002/smi.2654 26449710

[B112] Won J. Callow D. D. Pena G. S. Gogniat M. A. Kommula Y. Arnold-Nedimala N. A.. (2021). Evidence for exercise-related plasticity in functional and structural neural network connectivity. Neurosci. Biobehav. Rev. 131, 923–940. 10.1016/j.neubiorev.2021.10.013 34655658 PMC8642315

[B113] Zang Y. F. He Y. Zhu C. Z. Cao Q. J. Sui M. Q. Liang M.. (2007). Altered baseline brain activity in children with ADHD revealed by resting-state functional MRI. Brain Dev. 29, 83–91. 10.1016/j.braindev.2006.07.002 16919409

[B114] Zhang K. Jan Y.-. K Liu Y. Zhao T. Zhang L.. (2022). Exercise intensity and brain plasticity: what's the difference of brain structural and functional plasticity characteristics between elite aerobic and anaerobic athletes? Front. Hum. Neurosci. 16:757522. 10.3389/fnhum.2022.757522 35273485 PMC8901604

[B115] Zou Q. H. Zhu C. Z. Yang Y. Zuo X. N. Long X. Y. Cao Q. J.. (2008). An improved approach to detection of amplitude of low-frequency fluctuation (ALFF) for resting-state fMRI: Fractional ALFF. J. Neurosci. Methods 172, 137–141. 10.1016/j.jneumeth.2008.04.012 18501969 PMC3902859

[B116] Zwingmann L. Strütt S. Martin A. Volmary P. Bloch W. Wahl P.. (2019). Modifications of the Dmax method in comparison to the maximal lactate steady state in young male athletes. Phys. Sportsmed. 47, 174–181. 10.1080/00913847.2018.1546103 30408426

